# MosCoverY: A method to estimate mosaic loss of Y chromosome from sequencing coverage data

**DOI:** 10.1016/j.ajhg.2025.08.016

**Published:** 2025-09-11

**Authors:** Valeriia Timonina, Astrid Marchal, Laurent Abel, Aurélie Cobat, Jacques Fellay

**Affiliations:** 1 School of Life Sciences, École Polytechnique Fédérale de Lausanne, Lausanne, Switzerland; 2 Swiss Institute of Bioinformatics, Lausanne, Switzerland; 3 Laboratory of Human Genetics of Infectious Diseases, Necker Branch, INSERM U1163, Necker Hospital for Sick Children, Paris, France; 4 Imagine Institute, Université Paris Cité, Paris, France; 5 St. Giles Laboratory of Human Genetics of Infectious Diseases, Rockefeller Branch, The Rockefeller University, New York, NY, USA; 6 Biomedical Data Science Center, Lausanne University Hospital and University of Lausanne, Lausanne, Switzerland

**Keywords:** mosaic loss of the Y chromosome, mLOY, ageing, somatic mosaicism, exome sequencing

## Abstract

Mosaic loss of the Y chromosome (mLOY) is the most common somatic event in men, strongly associated with aging and various health conditions. Current methods for detecting mLOY primarily rely on DNA genotyping arrays. Here, we present MosCoverY, a method for estimating mLOY from exome or whole-genome sequencing data. MosCoverY addresses the challenges posed by the structure of the Y chromosome by focusing on single-copy genes and normalizing their coverage against autosomal exons matched by length and GC content. We validated it using data from 212,062 male participants in the UK Biobank, comparing the results to those obtained using genotyping- or whole-genome-sequencing-based methods. MosCoverY identified mLOY in 5.6% of men, demonstrating performance that was comparable to the other methods. We validated our approach by replicating known mLOY associations with age, smoking, all-cause mortality, and germline genetic loci. We further confirmed the robustness of our method at lower sequencing depth and demonstrated its applicability in single-sample analysis. Finally, we used data from The Cancer Genome Atlas to demonstrate that MosCoverY can also reliably detect variable mLOY in tumoral genomes. MosCoverY offers a valuable tool for detecting mLOY from exome or genome data in population-scale studies.

## Graphical abstract

**Figure:**
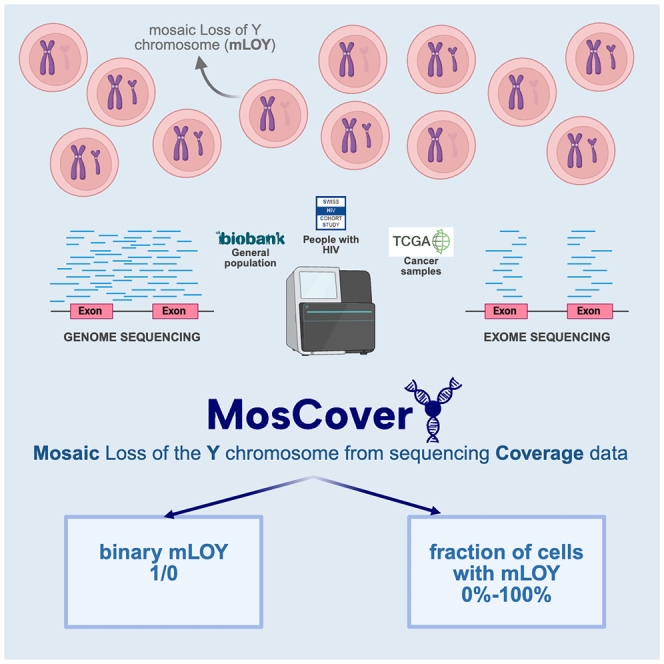

## Teaser

Mosaic loss of the Y chromosome (mLOY) is the most common somatic mutation in men. Its frequency increases with age, and it is associated with various diseases. Here, we present MosCoverY, a method that allows the identification and quantification of mLOY from exome and whole-genome sequencing data.

## Introduction

Somatic mutations are non-inheritable changes in the DNA of somatic cells leading to genetic mosaicism. These mutations are particularly well described in blood, where they can cause clonal hematopoiesis.^1^^,^^2^^,^^3^^,^^4^^,^^5^^,^^6^^,^^7^ The most commonly occurring somatic event in leukocytes of male individuals is the mosaic loss of the Y chromosome (mLOY).^8^^,^^9^^,^^10^^,^^11^^,^^12^ The prevalence of mLOY increases with age and is associated with various health-related conditions, including all-cause mortality,^13^^,^^14^ hematological cancer,^15^^,^^16^^,^^17^^,^^18^ solid tumors,^14^^,^^19^ cardiovascular diseases,^8^^,^^9^^,^^20^^,^^21^ and Alzheimer disease.^15^^,^^22^

mLOY can be detected molecularly using targeted quantitative PCR assay, genotyping arrays, or next-generation sequencing. The most commonly used methods utilize genotyping array data to detect the deviation in the intensity probe values on the Y chromosome (chrY). One such approach, mLRRY, calculates the median log-R ratio (mLRR; normalized intensity) in the male-specific part of chrY (MSY), excluding pseudoautosomal regions (PARs), and considers values below a chosen threshold as evidence of mLOY.^11^^,^^19^ Another method, PAR-LOY, uses long-range phasing information in the PARs to evaluate the allelic intensity imbalance in heterozygous sites between sex chromosomes.^23^

It is also possible to estimate mLOY from whole-genome sequencing (WGS) data using tools developed for copy-number variant calling in tumor cells, even if a normal sample is not available. One example is Control-FREEC, which calculates the ploidy of genomic regions from coverage data, taking into account GC content and genome mappability information.^24^^,^^25^ Although the mLOY estimation from Control-FREEC can be of high quality, WGS data are not always available and are computationally expensive to work with. Control-FREEC can be run on exome sequencing data, but this requires a matched normal sample, similar to other copy-number callers like FACETS.^8^^,^^26^

Currently, no method is available to call mLOY from exome sequencing data that can be applied to studies with only one sample per individual. Calling mLOY from exome sequencing data presents several challenges, such as limited coverage of the chrY (exome capture kits target mainly coding regions), biases in GC content that can affect coverage estimation, and the presence of repetitive sequences and regions highly similar to chrX on chrY.^27^^,^^28^

Here, we propose an approach to estimating mLOY from exome sequencing data that overcomes these challenges. We also show that our method can be directly used on WGS data. We demonstrate its performance by comparing it with two methods based on genotyping array data— mLRRY and PAR-LOY—as well as with one WGS-based method—Control-FREEC. Finally, we validated it using data from the UK Biobank (UKB), the Swiss HIV Cohort Study (SHCS), and The Cancer Genome Atlas (TCGA).

## Subjects and methods

### Study participants

We used data from three cohorts with available exome sequencing data—the UKB, SHCS, and TCGA.

The UKB is a large, prospective cohort study consisting of approximately 500,000 participants from the United Kingdom, with extensive phenotypic, genetic, and imaging data collected.^29^^,^^30^ The UKB data are available for research purposes upon project submission as described (https://www.ukbiobank.ac.uk/enable-your-research/apply-for-access). We selected participants from UKB who satisfied the following criteria: male genetic and self-reported sex, absence of sex aneuploidy, and availability of exome sequencing data. We further limited the choice of participants to those included in the Loh et al. study, which identified all mosaic chromosomal alterations (mCAs), including loss of chrY, and returned the data to the UKB (return #2062).^31^ After applying all the filters mentioned above, the total sample size was 212,062 individuals. For survival analyses and genome-wide association studies (GWASs), we included only individuals of European ancestry, reducing the sample size to 178,073.

The SHCS (www.shcs.ch) is a nationwide longitudinal cohort study enrolling adult people with HIV (PWH) living in Switzerland. The cohort study was established in 1988 and covers approximately 70% of all people ever diagnosed with HIV in Switzerland, with cumulatively more than 21,000 participants. In-depth clinical, demographic, lifestyle, and laboratory endpoints are collected in biannual follow-up visits.^32^ We here included a subset of 337 male individuals with available exome sequencing data.

From TCGA (https://gdc.cancer.gov/access-data/obtaining-access-controlled-data), a cancer genomics program that molecularly characterized over 20,000 primary cancers and matched normal samples spanning 33 cancer types, we selected 1,988 male individuals older than 40 years with primary tumors whose both normal and tumor samples were sequenced with Agilent’s Custom v.2 Exome Bait and who had available copy-number segments data called from Affymetrix SNP 6.0 genotyping array and WGS data.

### Ethics approval

The use of UKB data is covered by the generic ethical approval for UKB studies from the NHS National Research Ethics Service (ref. 11/NW/0382). The SHCS was approved by the ethics committees of all participating institutions (BASEC-Nr. 2023-02080). For TCGA, ethical approval was waived since we used publicly available open-access data.

### Selecting chrY exons from the exome capture kits

Due to the structure of the MSY, we restricted the choice of genes that were used to calculate chrY coverage. We excluded genes in the ampliconic part of chrY, as their copy number can vary a lot between individuals, and genes in X-transposed regions, due to their high similarity to the chrX sequences, which could affect coverage estimation. We included only single-copy genes in the X-degenerate regions of MSY (as described in Skaletsky et al.^27^), resulting in 13 genes (*SRY*, *RPS4Y1*, *ZFY*, *AMELY*, *TBL1Y*, *USP9Y*, *DDX3Y*, *UTY*, *TMSB4Y*, *NLGN4Y*, *KDM5D*, *EIF1AY*, and *RPS4Y2*).

For the IDT xGen Exome Research Panel v.1.0 used in the UKB, there were 184 exons in single-copy genes of chrY. We further filtered out exons that were outliers by coverage in individuals without evidence of mLOY as identified by the PAR-LOY method, reducing the number of exons to 173 (out of 45 genes with 578 exons captured).

The SHCS exomes were sequenced in 5 batches (BroadNeut, HIV_SC, HIV_VNP, SHCS392, and SystemX) using 3 different capture kits—SureSelect v.4 (batch SHCS392), SureSelect v.5 (batches BroadNeut, HIV_SC, and HIV_VNP), and xGen (batch SystemX). There were 164 and 215 exons in single-copy genes of chrY for SureSelect v.4 and SureSelect v.5, respectively. We focused on the same set of 173 exons as in the UKB for the sample sequenced with xGen.

In TCGA, various capture kits were used for exome sequencing. We focused on individuals sequenced with Agilent’s Custom v.2 Exome Bait, as it was the most common one. This kit captured 171 exons in single-copy genes of chrY.

### Selecting autosomal exons for normalization

For each selected exon on chrY, we found 100 autosomal exons matched by GC content and length (Figure S1). These autosomal exons were selected from the regions of the genome that are rarely gained or lost in blood (less than 10 gains and losses in the UKB database; return #2062)^33^ and matched by propensity score using the MatchIt R library. All matched exons for IDT xGen v.1, SureSelect v.4, SureSelect v.5, and Agilent’s Custom v.2 capture kits are listed in Tables S1, S2, S3, and S4.

### Computing normalized chrY coverage

**Figure 1:**
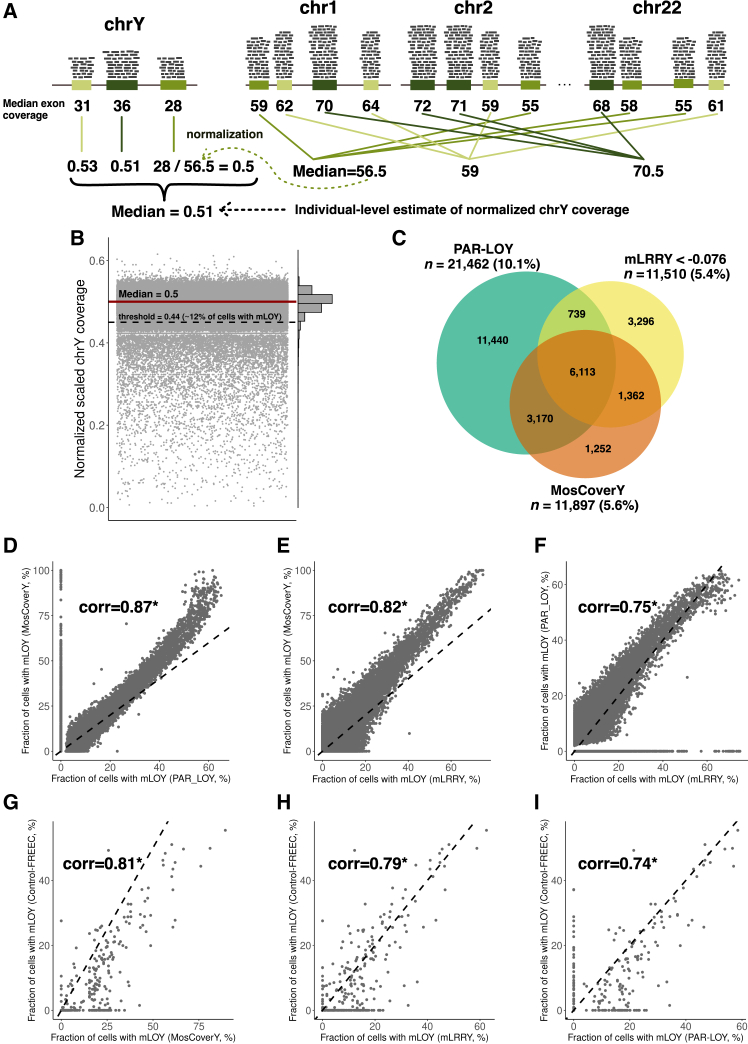
Description of the MosCoverY method and comparison with mLOY results obtained with mLRRY and PAR-LOY (A) One of the core principles of MosCoverY is the normalization of median coverage of selected chrY exons to a set of autosomal exons matched on GC content and length. Matched groups are represented by different shades of green. The individual-level estimation of chrY coverage corresponds to the median across all normalized chrY exon coverage values. (B) The distribution of normalized scaled chrY coverage in the UKB. Each dot represents an individual. The median of the distribution was scaled to 0.5 (red line), and the threshold to define mLOY was derived as Q1 − 1.5 × IQR and is equal to 0.44 (dashed black line). All individuals with normalized scaled chrY coverage below the threshold are considered mLOY carriers. (C) Venn diagram comparing MosCoverY results with two other mLOY calling methods—PAR-LOY and mLRRY—with an indication of the total number of mLOY carriers and their proportion according to each of the methods. (D–F) Correlation of the fraction of cells with mLOY derived from each of the three methods—MosCoverY vs. PAR-LOY, MosCoverY vs. mLRRY, and mLRRY vs. PAR-LOY. The black dashed line represents a diagonal (corr, Pearson’s correlation coefficient; ^∗^*p* ≤ 0.001). (G–I) Correlation of the fraction of cells with mLOY derived from MosCoverY, mLRRY, and PAR-LOY with cell fraction estimated from WGS with Control-FREEC for a subset of 360 randomly chosen individuals. The black dashed line represents a diagonal (^∗^*p* ≤ 0.001).

We calculated the median coverage of each of the selected exons (on chrY and autosomes) with the mosdepth tool from exome alignment files in CRAM format.^34^ We normalized the median coverage of each selected chrY exon to the median coverage of its 100 matched autosomal exons, and the median of the results was an individual-level estimate of normalized chrY coverage (Figure 1A). Normalization to matched autosomal exons significantly reduced noise and improved correlation with other methods compared to normalization to the median coverage of all exons on chr1 (Figure S2). The distribution of median chrY coverage in the population was then rescaled to 0.5 (the expected value given chrY’s haploid nature) by subtracting the population median of normalized chrY coverage and adding 0.5.

### Defining binary mLOY threshold

We defined a binary trait of mLOY by setting the threshold at Q1 − 1.5 × interquartile range (IQR) of the scaled chrY coverage distribution, which is a standard method for outlier detection.

### Converting scaled normalized chrY coverage to cell fraction

We converted the scaled normalized coverage of chrY to a fraction of cells with mLOY using the following formula (Figure S3):

(Equation 1) $$CF\;=\;\left\{\begin{array}{ll} 0 & if\;covY\;\geq 0.5\\ -2\times covY+1 & if\;0<covY<0.5\\ 1 & if\;covY\leq 0, \end{array}\right)$$

where CF is the fraction of cells with mLOY and covY is the normalized scaled coverage of the chrY.

### Other mLOY estimation methods in the UKB

To evaluate the performance of our method, we compared it to state-of-the-art methods used to estimate mLOY from genotyping or WGS data.

The first method uses mLRRY. We calculated mLRRY over 691 SNP probes in MSY and converted mLRRY to the fraction of cells with mLOY using the formula^25^

(Equation 2) $$CF=100\times \left(1-2^{2\times \text{mLRRY}}\right)\text{,}$$

where CF is the fraction of cells with mLOY and mLRRY is mLRR over genotyping probes on MSY.

We used the threshold of mLRRY < −0.076 to define the binary mLOY trait, corresponding to 10% of cells with mLOY as previously published.^13^

The second method, PAR-LOY, uses genotyping array probe intensity to define allelic imbalance in heterozygous sites of PARs of chrY.^23^ The PAR-LOY estimate is available as part of UKB return #2062 and provides both the binary mLOY estimates and the fraction of cells with mLOY (which is less than 10% for the majority of people).

For a random subset of 360 individuals, we estimated the relative chrY coverage with Control-FREEC (https://github.com/BoevaLab/FREEC) software.^24^^,^^35^ Control-FREEC segments the genome and identifies copy-number alterations using GC-content normalization and genome mappability information. We ran it on WGS alignment files and calculated the median ratio (normalized coverage) across all windows, excluding windows with a median ratio of more than one, as they likely represent repetitive regions of chrY and would add noise to the mLOY estimation. To convert it to the cell fraction, we used Equation 1, as for MosCoverY.

### Other mLOY estimation methods in TCGA

To estimate the performance of MosCoverY on the cancer dataset, we compared it to copy-number segmentation calls already available in TCGA (https://docs.gdc.cancer.gov/Data/Bioinformatics_Pipelines/CNV_Pipeline/). We utilized copy-number segment files generated from the Affymetrix SNP 6.0 genotyping array and WGS data. For Affymetrix SNP 6.0 genotyping array data, the DNACopy pipeline (https://bioconductor.org/packages/DNAcopy) was used to generate copy-number segment files, which associate contiguous chromosome regions with log2 ratio segment means. For WGS data, the GATK4 CNV pipeline (https://github.com/broadinstitute/gatk/tree/4.0.1.1/scripts/cnv_wdl) was used.

### Exome coverage downsampling

We performed a downsampling of UKB exome data using the GATK v.4.4.0.0 DownsampleSam tool (https://gatk.broadinstitute.org/hc/en-us/articles/360037056792-DownsampleSam-Picard) on a randomly selected set of 4,200 individuals. We applied it to the original CRAM files with the default parameters, setting the fraction of reads to keep to 0.1, 0.3, and 0.6 to keep 10%, 30%, and 60% of the reads, respectively.

### Epidemiological validation: Association of mLOY with age and smoking, as well as with all-cause mortality

To validate our method, we assessed the associations of mLOY with age and smoking status. We fitted a logistic regression model for the binary estimates of mLOY and a linear regression model for the fraction of cells with mLOY. We built separate models for each mLOY estimation method, fitting age at the first visit to the assessment center and ever-smoking status in the same model. We used the glm function with binomial family and the lm function from R statistical software (v.4.1.1) to fit logistic and linear models, respectively.

We also performed a survival analysis of all-cause mortality, modeling time to death using multivariable-adjusted Cox proportional hazard regression with age at the first visit to the assessment center, smoking status (current, previous, or never), and genetic principal components (PCs) as covariates. Time interaction terms for age and smoking status were included to satisfy the proportional hazards assumption of the Cox model. The target variable was the binary mLOY trait estimated by the various methods. We used the coxph function from the survival package (https://cran.r-project.org/web/packages/survival/index.html) of R statistical software (v.4.1.1).

### Genetic validation: GWAS

For the GWAS, we focused on men of European ancestry. We filtered out variants with minor-allele frequencies (MAFs) of less than 1%, minor-allele counts of less than 20, missing call frequencies greater than 0.1, and strong Hardy-Weinberg equilibrium deviation (p < 1e−15). Genetic PCs for the set of European-ancestry men were calculated with plink2 (https://www.cog-genomics.org/plink/2.0/) on an LD-pruned set of 149,605 SNPs using the snpgdsLDpruning function from the SNPRelate R package (https://www.bioconductor.org/packages/release/bioc/html/SNPRelate.html).

We performed genetic association testing with regenie (https://rgcgithub.github.io/regenie/)^36^ in two steps: whole-genome ridge regression to reduce the dimension of genetic data (611,713 variants), followed by association testing with a larger set of variants (9,607,831 imputed variants). Covariates included age, ever-smoking status, genotyping batch, WES release, and 10 genetic PCs.

We identified statistically independent signals with GCTA-COJO (genome-wide complex trait analysis multi-SNP-based conditional and joint association analysis using GWAS summary data; https://yanglab.westlake.edu.cn/software/gcta/#COJO).^37^^,^^38^ We used a *p* value threshold of 5e−8 to identify genome-wide significant SNPs, considering SNPs more than 1 Mb apart to be in linkage equilibrium and setting the collinearity threshold between SNPs to less than 0.9.

## Results

### Estimation of mLOY from UKB exome sequencing data

We developed an approach that we called MosCoverY (mosaic loss of chrY from sequencing coverage data) to estimate mLOY from exome sequencing or WGS data. MosCoverY uses alignment files (in BAM or CRAM format) and performs several steps to estimate the normalized median coverage of chrY. Key features of MosCoverY include focusing on single-copy genes on chrY and normalizing the coverage of exons in these genes to autosomal exons matched by GC content and length. This approach provides an individual-level estimate of normalized median chrY coverage, which is then scaled to match the expected population median of 0.5 (see subjects and methods for details). Of note, this scaling procedure adjusts the median of the normalized chrY coverage to the haploid value of 0.5, which is necessary for cell fraction estimation, but does not impact the relative ranking of samples or the outlier threshold used for binary mLOY calls. Using this normalized median coverage, we statistically derive a threshold for binary mLOY determination, defined as Q1 − 1.5 × IQR, following a standard outlier detection threshold. Samples with normalized scaled chrY coverage below this value are classified as mLOY carriers (Figure 1A).

We applied MosCoverY to the exomes of 212,062 men who contributed samples to the UKB. The distribution of normalized scaled chrY coverage is shown in Figure 1B (individuals with normalized scaled chrY coverage higher than 0.6 or lower than 0 are not shown). Using the binary threshold of 0.44 (derived as Q1 − 1.5 × IQR), MosCoverY identified 11,897 (5.6%) mLOY carriers. We converted the scaled normalized coverage of chrY to a fraction of cells with mLOY using Equation 1 (subjects and methods). The mean fraction of cells with mLOY estimated by MosCoverY in all UKB participants was 2.8% (including individuals without mLOY).

In comparison, binary estimates based on mLRRY and PAR-LOY identified 11,510 (5.4%) and 21,462 (10.1%) mLOY carriers, respectively. Across all three methods, mLOY was identified in 6,113 men (22.3% of all mLOY carriers; Figure 1C). Unique to MosCoverY, 1,252 mLOY carriers (4.6% of the union of all mLOY carriers) were identified, compared to 3,296 (12%) and 11,440 (41.8%) identified solely by mLRRY and PAR-LOY, respectively. Normalized scaled coverage of chrY estimated by MosCoverY was lower for mLOY carriers identified by MosCoverY only, compared to those identified by either mLRRY or PAR-LOY or by both, but it was higher compared to mLOY carriers identified by MosCoverY and either mLRRY or PAR-LOY. mLOY carriers identified by all three methods had the lowest normalized scaled coverage of chrY (Figure S4 shows the normalized scaled coverage of chrY for different intersection groups in Figure 1C).

The mean fractions of cells with mLOY estimated by mLRRY and PAR-LOY were 2.4% and 1.3%, respectively, both lower than the 2.8% estimated by MosCoverY. The fraction of cells with mLOY estimated by MosCoverY showed a strong correlation with both mLRRY and PAR-LOY estimates (Pearson’s correlation coefficient > 0.8; Figures 1D and 1E), which was higher than the correlation between mLRRY and PAR-LOY estimates (Pearson’s correlation coefficient = 0.75; Figure 1F). We also estimated the fraction of cells with mLOY from WGS data with the Control-FREEC tool^24^^,^^35^ for a subset of 360 randomly chosen individuals. The MosCoverY-derived fraction of cells with mLOY showed better correlation with the Control-FREEC-derived cell fraction as compared to mLRRY and PAR-LOY (Figures 1G–1I).

Although our primary goal was to develop a method for identifying mLOY from exome sequencing data, MosCoverY can also be applied directly to WGS data. We demonstrated this by applying MosCoverY to a subset of 4,200 randomly selected individuals with WGS data. The normalized coverage of chrY estimated from exome sequencing and WGS showed a strong correlation (Pearson’s correlation coefficient = 0.9). Furthermore, 74% of individuals identified as mLOY carriers were consistent between exome sequencing and WGS (Figure S5). Additionally, exome sequencing and WGS data can be combined for mLOY identification. However, in this case, they should be scaled separately before combining to define the mLOY threshold rather than being combined prior to scaling (compare Figures S6A and S6B). mLOY estimated from WGS data with MosCoverY also correlated well with mLOY estimated from WGS data with Control-FREEC in a subset of 360 individuals (Pearson’s correlation coefficient = 0.72 for normalized scaled coverage and 0.82 for the fraction of cells with mLOY; Figure S7).

### Effect of coverage on mLOY estimation accuracy

To check the robustness of MosCoverY’s results to fluctuations in exome sequencing coverage, we compared its performance for various fractions of the original coverage—60%, 30%, and 10% of aligned reads. We performed the downsampling of the original CRAM files for 4,200 randomly selected individuals from the UKB. The estimated normalized chrY coverage was highly correlated between the downsampled and original CRAM files, although the correlation decreased with the decreasing fraction of reads (Figure S8).

### Epidemiological validation of MosCoverY in the UKB

In the absence of a gold-standard method to assess the presence of mLOY, we aimed to validate the performance of our approach by examining and comparing the strength of previously reported associations between mLOY and factors such as age, smoking, and all-cause mortality.

**Figure 2:**
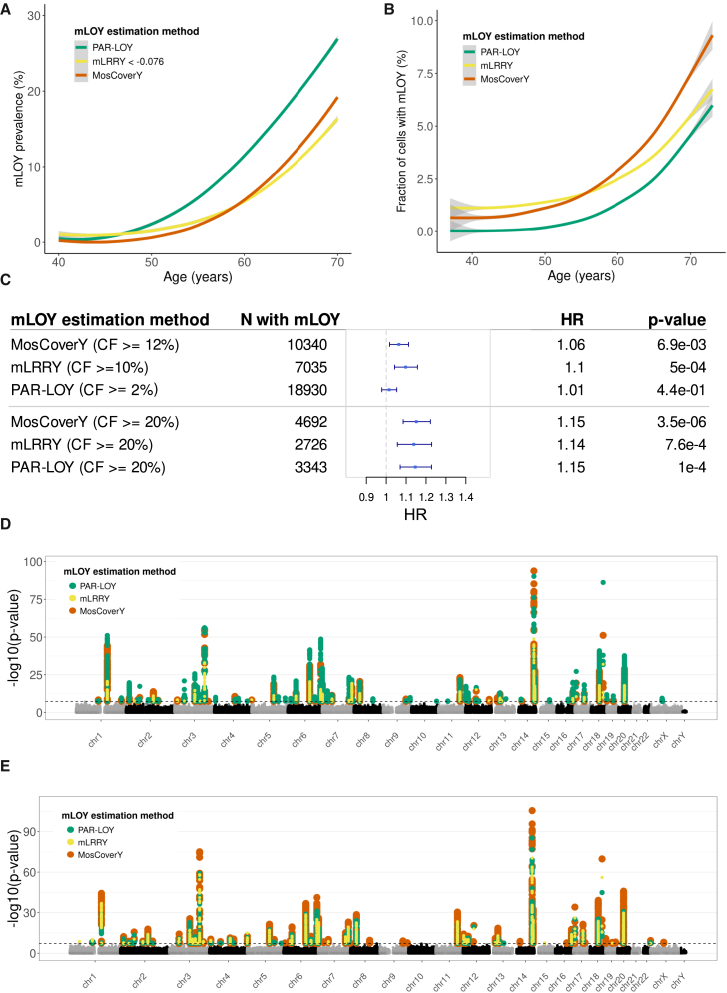
Biological validation of the MosCoverY method (A) Age-dependent prevalence of mLOY estimated by three methods, PAR-LOY, mLRRY, and MosCoverY, fit with local polynomial regression fitting. Shaded areas represent 95% confident intervals. (B) Age-dependent increase in the fraction of cells with mLOY estimated by three methods, PAR-LOY, mLRRY, and MosCoverY, fit with the generalized additive model. Shaded areas represent 95% confident intervals. (C) Association of binary mLOY trait with all-cause mortality estimated by Cox proportional hazard model adjusted for age, smoking status, and genetic PCs. The analysis is performed on individuals of European ancestry. CF, fraction of cells with mLOY; HR, hazard ratio. (D and E) Manhattan plot showing genetic loci associated with binary mLOY trait (D) and fraction of cells with mLOY (E). Each dot represents the genomic position, and the black horizontal dashed line represents a significant *p* value threshold (5e−8).

The prevalence of mLOY in the UKB, as estimated by all three methods, significantly increases with age (Figure 2A; Table 1). While the PAR-LOY method indicated the highest proportion of mLOY carriers (around 27% at age 70), the strongest associations with age and smoking and the best goodness of fit (residual deviance) were observed with the MosCoverY estimate according to logistic regression analyses (Table 1).

**Table 1:** Association of mLOY binary trait estimated by three methods with age and smoking

	**Beta**	**SE**	***p* value**	**Residual deviance**
**Association with age**				

MosCoverY	0.17	0.002	<1e−300	7.9e+04
PAR-LOY	0.14	0.001	<1e−300	1.2e+05
mLRRY < −0.076	0.12	0.002	<1e−300	8.1e+04

**Association with ever-smoking status**				

MosCoverY	0.48	0.02	2.7e−96	7.9e+04
PAR-LOY	0.32	0.02	2.2e−77	1.2e+05
mLRRY < −0.076	0.37	0.02	7.4e−61	8.1e+04

The fraction of cells with mLOY was also positively associated with age and smoking (Figure 2B; Table 2), with the MosCoverY estimates again showing the strongest association and highest R-squared value.

**Table 2:** Association of fraction of cells with mLOY estimated by three methods with age and smoking

	**Beta**	**SE**	***p* value**	**Adjusted R-squared**
**Association with age**				

MosCoverY CF	0.2	0.002	<1e−300	0.065
PAR-LOY CF	0.13	0.001	<1e−300	0.048
mLRRY CF	0.13	0.001	<1e−300	0.045

**Association with ever-smoking status**				

MosCoverY CF	0.7	0.03	2.1e−116	0.065
PAR-LOY CF	0.47	0.02	8.0e−90	0.048
mLRRY CF	0.44	0.02	3.6e−81	0.045

We further assessed the previously reported association of mLOY with all-cause mortality.^39^ Using a Cox proportional hazards model, we evaluated time to death with mLOY binary traits as explanatory variables, adjusting for age, smoking status, and genetic PCs. Binary mLOY estimates from MosCoverY and mLRRY were significantly associated with all-cause mortality, with mLRRY showing a slightly stronger association. In contrast, the PAR-LOY estimates were not associated with all-cause mortality, likely because most mLOY carriers identified by PAR-LOY had mLOY in fewer than 10% of cells. Higher fractions of cells with mLOY are known to be associated with a greater risk of death.^20^ When defining the binary mLOY trait by a higher cell fraction (≥20%), we observed stronger associations with all-cause mortality across all three methods, with MosCoverY showing the strongest association (Figure 2C).

In addition, we compared the association of mLOY with age and smoking across different groups of mLOY carriers identified by one, two, or all three methods (as depicted in Figure 1C). The intersection of mLOY carriers identified by all three methods (*n* = 6,113) showed the strongest association with both age and smoking. Among the mLOY carriers identified by the two methods, the group identified by MosCoverY and mLRRY (*n* = 1,362) showed the next strongest association. In the groups identified by only one of the methods, MosCoverY (*n* = 1,252) demonstrated the strongest association with age and smoking, followed by PAR-LOY (*n* = 11,440; Table S5). When assessing the association of mLOY with all-cause mortality in the same groups (but in the subset of European ancestry individuals), the only significant association was in the group of mLOY carriers identified by all three methods (Figure S9).

### Genetic validation of MosCoverY in the UKB

Previously, many genetic loci have been identified as associated with mLOY, estimated with mLRRY and PAR-LOY, through GWASs.^10^^,^^23^ To better compare the strength of these known genetic associations for three mLOY estimates, we repeated the GWAS on the set of 178,073 UKB participants of European ancestry for mLOY traits estimated by mLRRY and PAR-LOY and compared the results to MosCoverY. We performed separate GWASs for the binary mLOY trait (Figure 2D) and for the fraction of cells with mLOY (Figure 2E).

We identified 36, 50, and 23 independent genetic signals for binary mLOY calls from MosCoverY, PAR-LOY, and mLRRY, respectively. A total of 22 loci were shared between all three methods: 31 between MosCoverY and PAR-LOY, 23 between MosCoverY and mLRRY, and 22 between PAR-LOY and mLRRY. The strongest association identified by all methods (rs56349439 at chr14:101168739) had the smallest *p* value for MosCoverY (*p* = 6.84e−24 for MosCoverY vs. 1.86e−18 and 4.05e−13 for PAR-LOY and mLRRY, respectively). All SNPs identified as independently associated with binary mLOY traits are listed in Tables S6, S7, and S8.

We identified 59, 35, and 38 loci associated with the fraction of cells with mLOY, as estimated by MosCoverY, PAR-LOY, and mLRRY, respectively. A total of 31 loci were shared between all three methods: 34 between MosCoverY and PAR-LOY, 36 between MosCoverY and mLRRY, and 32 between PAR-LOY and mLRRY. Most of the genetic signals associated with the fraction of cells with mLOY intersected with those associated with the binary mLOY trait, and most showed the strongest associations for MosCoverY estimates (Figure 2E). All SNPs identified as independently associated with the fraction of cells with mLOY traits are listed in Tables S9, S10, and S11.

### Application to TCGA

To further validate our approach, we applied it to an independent dataset from TCGA.^32^ Specifically, we analyzed both normal and tumor samples from 1,988 men with primary tumors, using exome sequencing data generated with Agilent’s Custom v.2 Exome Bait.

In normal samples, mLOY was identified in 155 individuals (7.8%) using a binary threshold of 0.42, corresponding to approximately 16% of cells exhibiting mLOY (Figure S11A). The prevalence of mLOY increased with age (Figure S10). Accordingly, the normalized scaled coverage of chrY was negatively associated with age (Pearson’s correlation coefficient = −0.3, *p* < 2.2 × 10e−16).

In tumor samples, the distribution of normalized chrY coverage was broader than in normal samples. As the statistically derived threshold (0.03) was deemed too low, we instead applied an empirical threshold of 0.45, corresponding to ∼10% of cells with mLOY (Figure S11B). Using this threshold, mLOY was detected in 751 individuals (38%). The frequency of mLOY varied substantially across tumor types and tissues of origin, ranging from 2% in papillary thyroid adenocarcinoma to 89% in papillary kidney adenocarcinoma (Figure S12A). The average fraction of cells with mLOY in tumor samples was 18%, with values ranging from 0.7% in anaplastic cerebrum astrocytoma to 57% in papillary kidney adenocarcinoma (Figure S12B). The mean cell fraction was strongly correlated with mLOY prevalence across tumor types (Pearson’s correlation coefficient = 0.93, *p* < 2.2 × 10e−16), and both metrics were positively associated with the mean age of individuals within each tumor type and tissue category (Pearson’s correlation coefficients = 0.52 and 0.51; *p* = 0.001 and 0.002 for mLOY frequency and mean fraction of cells with mLOY, respectively). While normalized scaled chrY coverage remained significantly associated with age (Pearson’s correlation coefficient = −0.15, *p* = 4.524 × 10e−12), this relationship was weaker than in normal samples and varied across tumor types. A significant age association was observed only in clear cell kidney adenocarcinoma (Pearson’s correlation coefficient = −0.24, *p* = 0.02). Although mLOY prevalence increased with age, this trend was not consistent across all tumor types (Figure S10).

We also assessed the concordance of mLOY status between normal and tumor samples (Figure S11C). The correlation of normalized scaled chrY coverage between matched normal and tumor samples was modest (Pearson’s correlation coefficient = 0.12, *p* = 1.59 × 10e−7). In terms of binary mLOY status, 84 mLOY carriers were unique to normal samples, 680 to tumor samples, and 71 were found in both, yielding a Fisher’s exact test odds ratio of 1.4 (*p* = 0.04).

To validate the mLOY estimates produced by MosCoverY, we compared them to TCGA copy-number segment data derived from SNP arrays and WGS. Both normalized scaled chrY coverage and fraction of cells with mLOY were strongly correlated with these reference data, exceeding even the internal concordance between SNP array- and WGS-derived copy-number segment calls (Figure S13).

Finally, we tested two normalization strategies to potentially improve MosCoverY’s performance: excluding autosomal genes frequently affected by CNVs in each tumor type and normalizing chrY coverage to autosomal exons from matched normal samples. However, neither approach enhanced the performance; in fact, both reduced the correlation between normalized chrY coverage and TCGA copy-number segment data (data not shown).

### Single-sample analysis using SHCS data

Since MosCoverY relies on a cohort of individuals to estimate the threshold from the distribution of normalized scaled chrY coverage, we investigated whether the threshold identified from UKB participants could be directly applied to other populations. This would enable mLOY estimation for a single individual without the need for a cohort.

To explore this, we applied the method to 337 male participants from the SHCS, whose exome sequencing data were generated in 5 batches using 3 different capture kits: SureSelect v.4 (batch SHCS392), SureSelect v.5 (batches BroadNeut, HIV_SC, and HIV_VNP), and IDT xGen v.1 (batch SystemX). Coverage differed across capture kits but showed no significant dependence on sequencing batch (Figure S14A). Due to this variation, applying a uniform threshold across all batches without adjusting the normalized chrY coverage would be inappropriate (Figure S14B).

The SystemX batch was sequenced using the IDT xGen v.1 exome capture kit, identical to that used in the UKB. For this batch, we compared mLOY detection results using both the UKB-derived binary threshold and a threshold derived from the SHCS SystemX batch. In the UKB, the median normalized chrY coverage before scaling was 0.58, with a binary mLOY threshold set at 0.52. In the SHCS SystemX batch, the corresponding median was 0.59, and the binary mLOY threshold was 0.54. Applying the UKB-derived threshold identified 2 mLOY carriers (2.2%), while the SHCS-derived threshold identified 4 mLOY carriers (4.3%) (Figure S15A).

To estimate the fraction of cells with mLOY, the normalized chrY coverage must be rescaled. We compared the impact of rescaling using the UKB vs. SHCS median values. Specifically, we applied the transformation chrYcov − median + 0.5 and then used Equation 1 to convert the rescaled coverage into an estimated fraction of cells with mLOY. The estimates were highly correlated (Pearson’s correlation coefficient = 0.99, *p* < 2e−16), though slightly lower when the UKB median was used for rescaling (Figure S15B).

## Discussion

In this study, we developed MosCoverY, an approach to estimate mLOY from exome sequencing and WGS data. This method provides an individual-level estimate of normalized median chrY coverage, which is used to derive a binary mLOY estimate and a quantitative measure of the fraction of cells with mLOY. We demonstrated the robustness of the MosCoverY method by comparing it to two widely used mLOY estimation methods based on genotyping array data—mLRRY and PAR-LOY—using the UKB data. Our results demonstrate that MosCoverY provides a biologically meaningful estimate of mLOY, successfully overcoming the challenges of the chrY structure by accounting for gene copy number, homology with the chrX, and GC bias.

MosCoverY’s results showed a strong correlation with mLOY estimates from mLRRY and PAR-LOY. The highest number of mLOY carriers was identified by PAR-LOY, which can be explained by the relatively low threshold for the fraction of cells with mLOY used by default by this method: 2% vs. 10% and 12% for mLRRY and MosCoverY, respectively. Of note, mLOY identified only by PAR-LOY was not associated with all-cause mortality, suggesting the limited phenotypic relevance of mLOY with a low cell fraction. Some individuals were identified as mLOY carriers by mLRRY and MosCoverY but not by PAR-LOY (Figures 1D and 1F). This could be explained by the fact that PAR-LOY only uses data from the PARs, which represent a small fraction of the chrY, potentially resulting in false negative calls.

Defining the binary mLOY phenotype requires selecting a threshold, which we approached in a statistically defined manner. However, an alternative approach involves setting an arbitrary threshold based on a predefined fraction of cells with mLOY (Figure S16). As the threshold increases, the proportion of mLOY carriers identified by all three methods rises, relative to the total number of mLOY carriers identified by any method. Setting a higher threshold for the fraction of cells with mLOY may be appropriate in certain contexts, as a higher fraction of cells affected by mLOY is likely to have a more pronounced impact on health-related outcomes (like all-cause mortality in Figure 2C). Setting an arbitrary threshold can also be more appropriate when the expected number of mLOY carriers is high, which leads to a wider chrY coverage distribution and a lower statistically derived threshold and, as a consequence, many false negative calls. We demonstrated it in the case of tumor samples in TCGA.

The fraction of cells with mLOY also correlated well between the three methods, with the highest correlation coefficient between MosCoverY and PAR-LOY and the lowest for PAR-LOY and mLRRY (Figures 1D–1F). MosCoverY also showed the highest correlation with the Control-FRECC-estimated fraction of cells with mLOY from WGS data (Figures 1G–1I). Despite a good correlation, the MosCoverY-estimated cell fraction was, on average, higher compared to the two other methods, which can mean either overestimation of the fraction of cells with mLOY by MosCoverY or underestimation by PAR-LOY and mLRRY. Both possibilities cannot be confidently ruled out. One of the steps in MosCoverY is the rescaling of the normalized chrY coverage to match the median of the distribution to the expected value of 0.5, which might be a source of overestimation. The argument against it is the fact that the correlation of the fraction of cells with mLOY with other methods, when it is estimated from rescaled coverage values, improves compared to the cell fraction estimated from the original normalized coverage of chrY.

We also validated MosCoverY by replicating known associations between mLOY and various clinical, epidemiological, and genetic measurements. As expected, the prevalence of mLOY showed a massive positive correlation with age, and significant associations were observed with smoking status and all-cause mortality. These findings are consistent with previous studies, underscoring the relevance of our method. Notably, MosCoverY’s estimates showed the strongest associations with age and smoking, suggesting excellent sensitivity and robustness. Additionally, MosCoverY’s ability to identify germline genetic loci associated with mLOY, often showing stronger associations (especially for the quantitative fraction of cells with mLOY rather than the binary mLOY trait), further supports its accuracy and utility in genetic studies.

In addition to the UKB, we successfully applied MosCoverY to the exomes from 337 SHCS participants, as well as to normal and tumor exome sequencing data from 1,988 TCGA individuals, which provides additional evidence of the robustness of our method. Samples from these two cohorts were sequenced with different capture kits, and the results of MosCoverY showed expected patterns, such as mLOY association with age in SHCS and non-tumoral TCGA samples, and higher prevalence of mLOY in tumors compared to matching normal samples. These results also demonstrate the usability of MosCoverY for cancer studies, even if only tumor samples are available, since the estimated mLOY fractions showed high correlation with copy-number segment data available in TCGA, which were obtained using both tumor and normal samples. The prevalence of mLOY was found to vary across tumor types, consistent with a previous study that used the FACETS pipeline^8^^,^^26^ and chrY gene expression analysis for mLOY detection in male participants of TCGA.^8^

While MosCoverY effectively normalizes coverage against matched autosomal exons, discrepancies in GC content or other biases could still impact the results. Importantly, the exon-matching procedure needs to be repeated for each new exome capture kit.

Finally, the median of normalized chrY coverage and the binary mLOY threshold derived from the UKB data can be used in a case when only one sample is available, but only if it was sequenced with the same capture kit, that is, IDT xGen Exome Research Panel v.1.0, as we demonstrated for the SystemX batch of SHCS. But because the distribution of normalized chrY coverage varied between different capture kits, it is recommended to utilize a cohort of individuals to derive those metrics. This variation also highlights the relevance of rescaling the values to the expected median of 0.5.

MosCoverY can be directly applied to cohorts with exome sequencing and WGS data, offering a valuable tool for large-scale epidemiological studies of mLOY, the most common type of somatic, age-related DNA change. mLOY is part of a bigger group of somatic DNA changes called mCAs. Apart from mLOY, they include losses and gains of parts of or whole chromosomes, as well as copy-neutral loss of heterozygosity. All mCAs increase in prevalence with age, and many are associated with clonal hematopoiesis and various health-related conditions, such as hematological cancers,^40^ solid tumors, diverse types of infections,^4^ and dementia.^41^

Understanding the prevalence and impact of mLOY can provide insights into age-related diseases and male-specific health risks and can potentially help to explain the longevity gap between men and women, as was proposed in several studies.^14^^,^^42^^,^^43^

## Data and code availability

MosCoverY is available on GitHub at https://github.com/ValeraKus/mLOY_Exomes.

## Acknowledgments

This research was conducted using the UK Biobank Resource under application #84415. The results published here are in part based upon data generated by TCGA Research Network: https://www.cancer.gov/tcga. Funding for this work comes from 10.13039/501100001703EPFL, the 10.13039/501100001711Swiss National Science Foundation (grant #197721), the 10.13039/501100001665French National Research Agency (grant GENVIR ANR-20-CE93-003), and the 10.13039/501100000780European Commission through the Horizon Europe project UNDINE (grant ID: 101057100). This study has also been financed within the framework of the Swiss HIV Cohort Study (SHCS), supported by the Swiss National Science Foundation (grant #33FI-0_229621), by SHCS project #876, and by the SHCS Research Foundation.

## Author contributions

All authors contributed to the method development, study design, and preparation of the manuscript. V.T. performed method tests and analyses with UK Biobank, SHCS, and TCGA data.

## Declaration of interests

The authors declare no competing interests.

## Declaration of generative AI and AI-assisted technologies in the writing process

During the preparation of this work, the authors used ChatGPT in order to improve the text of the manuscript. After using this tool, the authors reviewed and edited the content as needed and take full responsibility for the content of the publication.

## Supplemental information

Document S1. Figures S1–S16

Table S1. All exons used to calculate normalized chrY coverage with normalizing groups (IDT xGen Exome Research Panel v.1.0)

Table S2. All exons used to calculate normalized chrY coverage with normalizing groups (Sure Select v.4)

Table S3. All exons used to calculate normalized chrY coverage with normalizing groups (Sure Select v.5)

Table S4. All exons used to calculate normalized chrY coverage with normalizing groups (Agilent’s Custom v.2 Exome Bait)

Table S5. Results of the multiple logistic regression analysis of mLOY cases defined by the intersection of different methods

Table S6. Independent SNPs associated with binary mLOY trait identified with MosCoverY

Table S7. Independent SNPs associated with binary mLOY trait identified with PAR-LOY

Table S8. Independent SNPs associated with binary mLOY trait identified with mLRRY

Table S9. Independent SNPs associated with quantitative fraction of cells with mLOY identified with MosCoverY

Table S10. Independent SNPs associated with quantitative fraction of cells with mLOY identified with PAR-LOY

Table S11. Independent SNPs associated with quantitative fraction of cells with mLOY identified with mLRRY

Document S2. Article plus supplemental information
