# Supplementary material for: MosCoverY: A method to estimate mosaic loss of Y chromosome from sequencing coverage data
Source: Am J Hum Genet. 2025 Sep 11;112(10):2509–19. doi: 10.1016/j.ajhg.2025.08.016 (PMC12696490; doi:10.1016/j.ajhg.2025.08.016)
Supplement: Document S1. Figures S1–S16 [file mmc1.pdf]

The American Journal of Human Genetics, Volume *112*

Supplemental information

MosCoverY: A method to estimate mosaic loss of Y chromosome from sequencing coverage data

Valeriia Timonina, Astrid Marchal, Laurent Abel, Aurélie Cobat, and Jacques Fellay

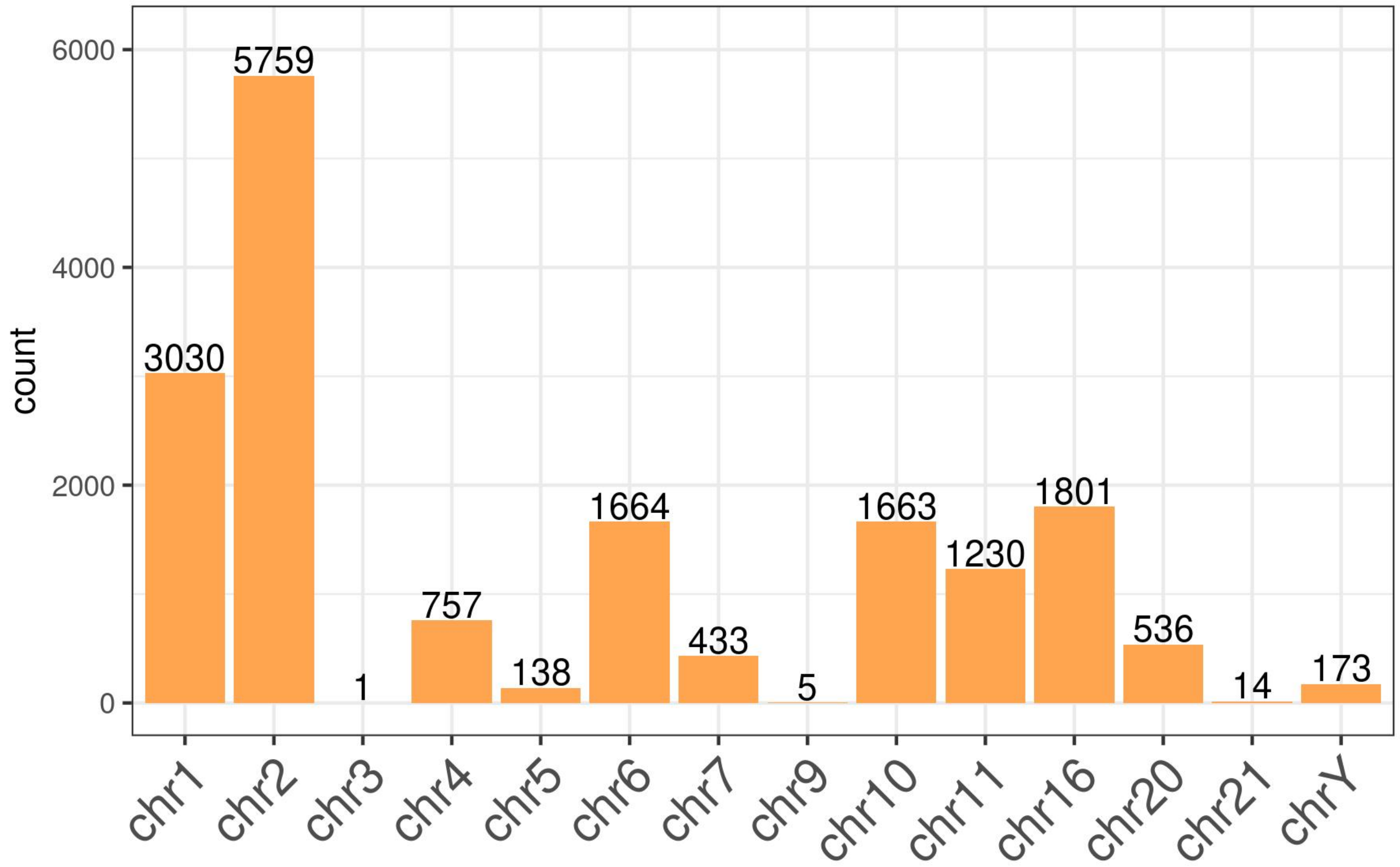

count
6000
4000
2000
0
5759
3030
1
757
138
1664
433
5
1663
1230
1801
536
14
173
chr1
chr2
chr3
chr4
chr5
chr6
chr7
chr9
chr10
chr11
chr16
chr20
chr21
chrY

Figure S1. Number of exons on each chromosome selected for the MosCoverY when used with the xGen exome capture kit: for each of the 173 exons on chrY, we selected 100 exons matched by length and GC-content on autosomes for the coverage normalization.

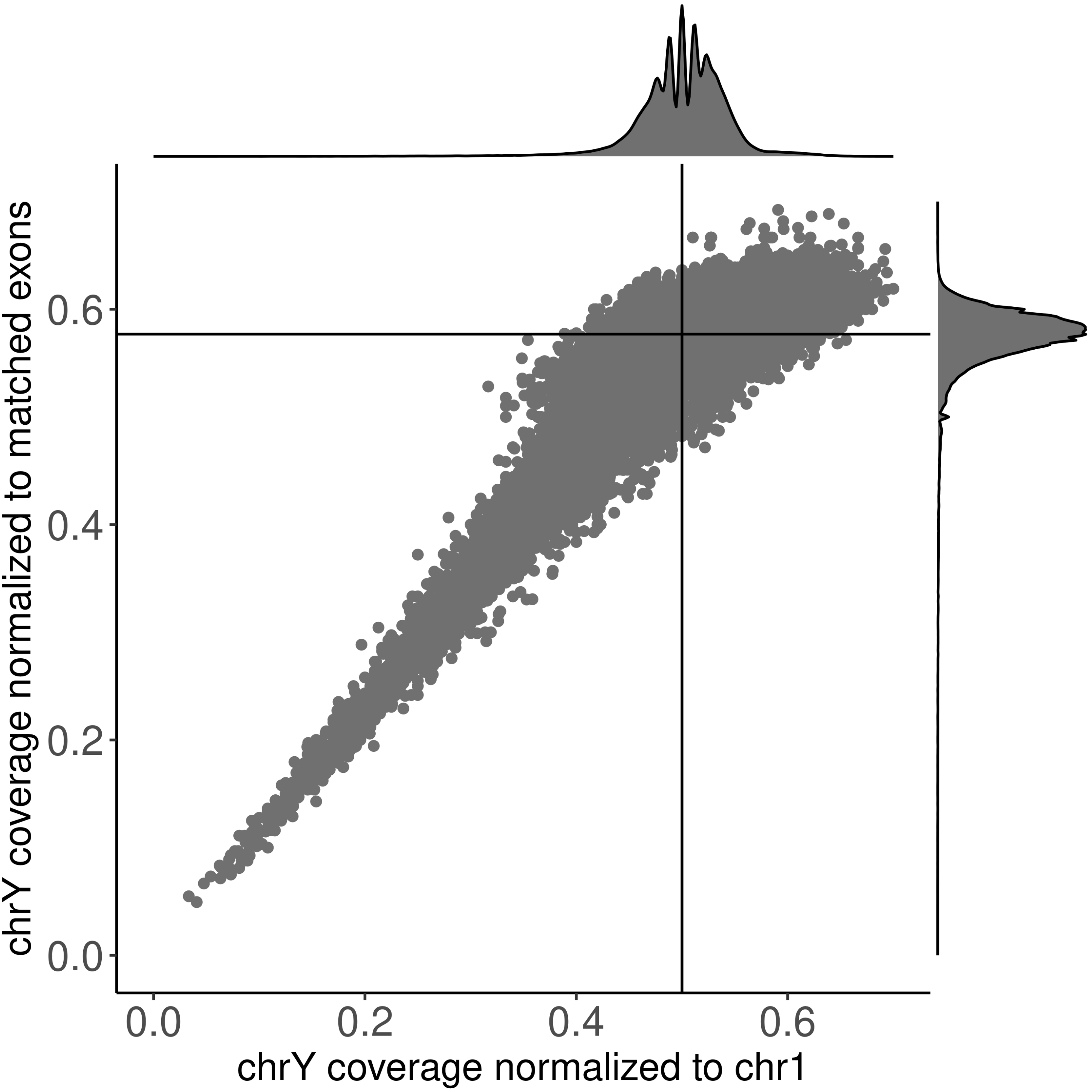

chrY coverage normalized to matched exons
0.0
0.2
0.4
0.6
0.0
0.2
0.4
0.6
chrY coverage normalized to chr1

Figure S2. Comparison of individual-level estimates of normalized chrY coverage when normalizing on matched autosomal exons (Fig. S1) and on all exons on chr1. The horizontal and vertical lines indicate the medians for the two estimates.

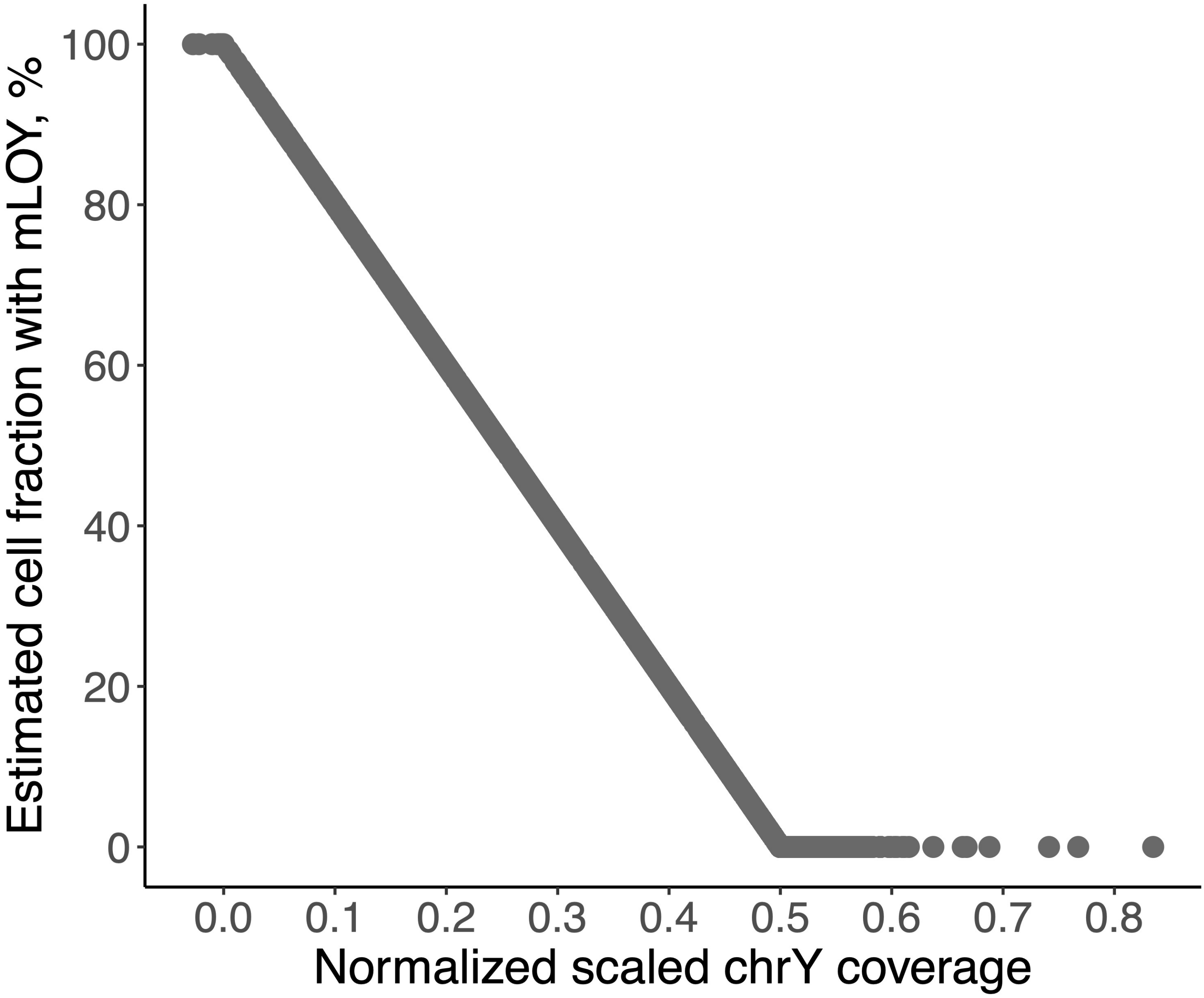

Estimated cell fraction with mLOY, %
100
80
60
40
20
0
0.0
0.1
0.2
0.3
0.4
0.5
0.6
0.7
0.8
Normalized scaled chrY coverage

Figure S3. Conversion of normalized scaled chrY coverage (x-axis) from MosCoverY to the fraction of cells with mLOY (y-axis) using Equation 1 (see Subjects and Methods).

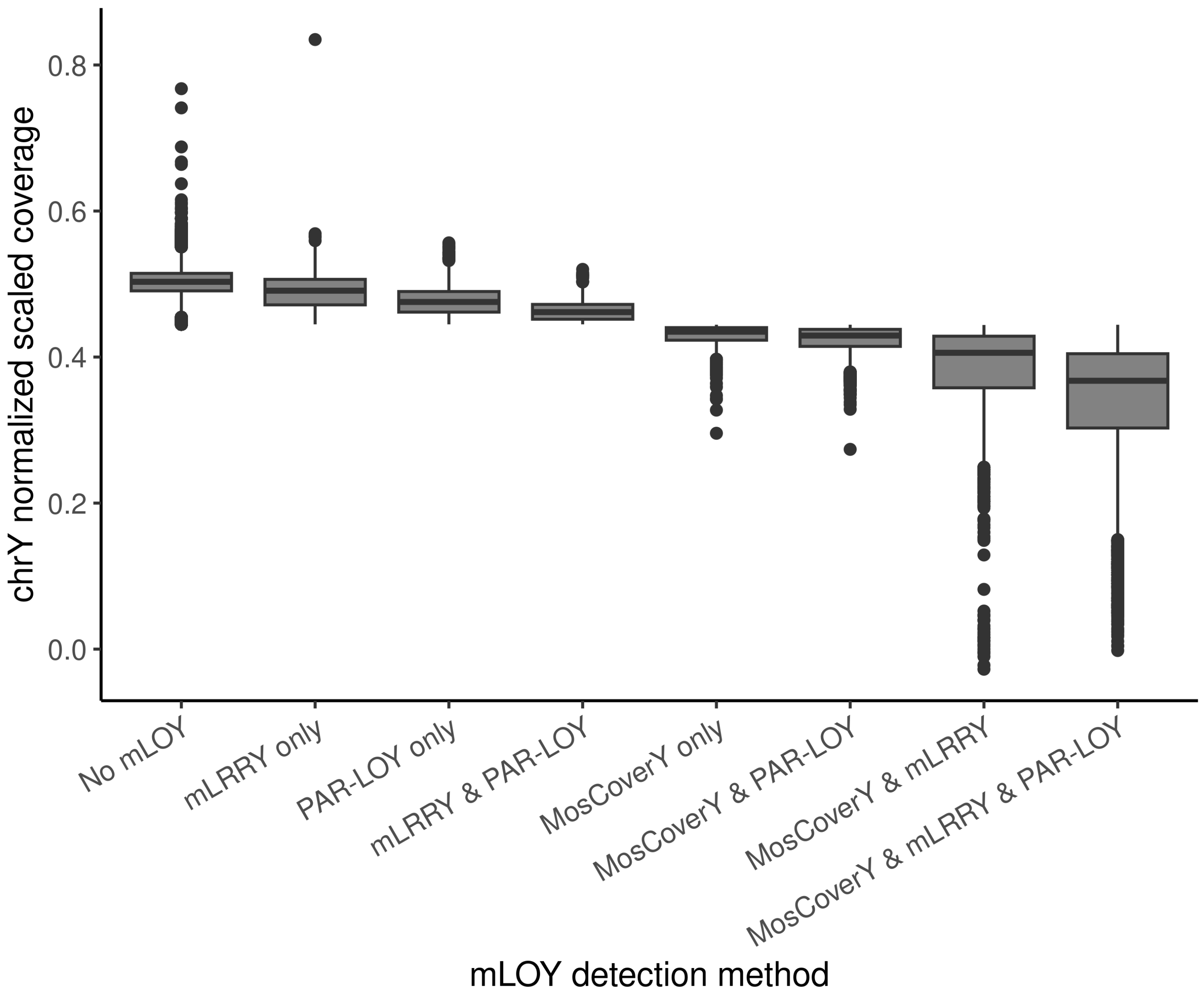

chrY normalized scaled coverage
0.8
0.6
0.4
0.2
0.0
No mLOY
mLRRY only
PAR-LOY only
mLRRY & PAR-LOY
MosCoverY only
MosCoverY & PAR-LOY
MosCoverY & mLRRY
MosCoverY & mLRRY & PAR-LOY
mLOY detection method

Figure S4. Normalized scaled coverage of chrY estimated with MosCoverY from exome sequencing data in the UKB in groups of mLOY carriers as defined uniquely by one of the three methods (PAR-LOY, mLRRY, and MosCoverY) and their intersections. Groups are the same as in Fig. 1C. “No mLOY” represents individuals without mLOY as identified by all three methods.

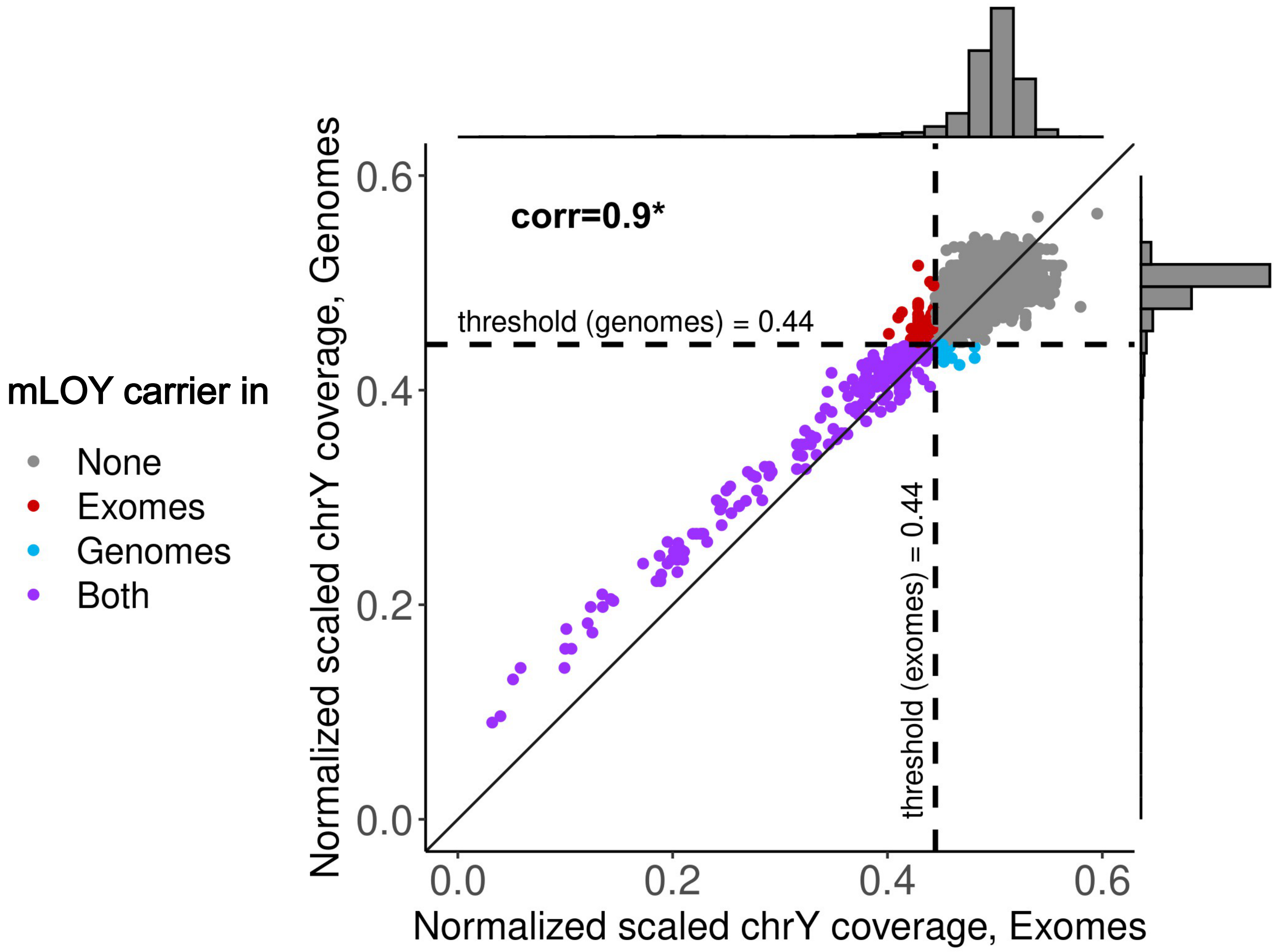
mLOY carrier in
None
Exomes
Genomes
Both
corr=0.9*
threshold (genomes) = 0.44
threshold (exomes) = 0.44
0.0
0.2
0.4
0.6
Normalized scaled chrY coverage, Genomes
Normalized scaled chrY coverage, Exomes

Figure S5. Comparison of applying MosCoverY to the exome sequencing and WGS data of 4,200 randomly selected participants from the UKB. The thresholds are defined as Q1−1.5×IQR of normalized scaled chrY coverage for genomes and exomes separately and are shown as horizontal and vertical dashed lines (corr - Pearson’s correlation coefficient, * - p-value < 0.001).

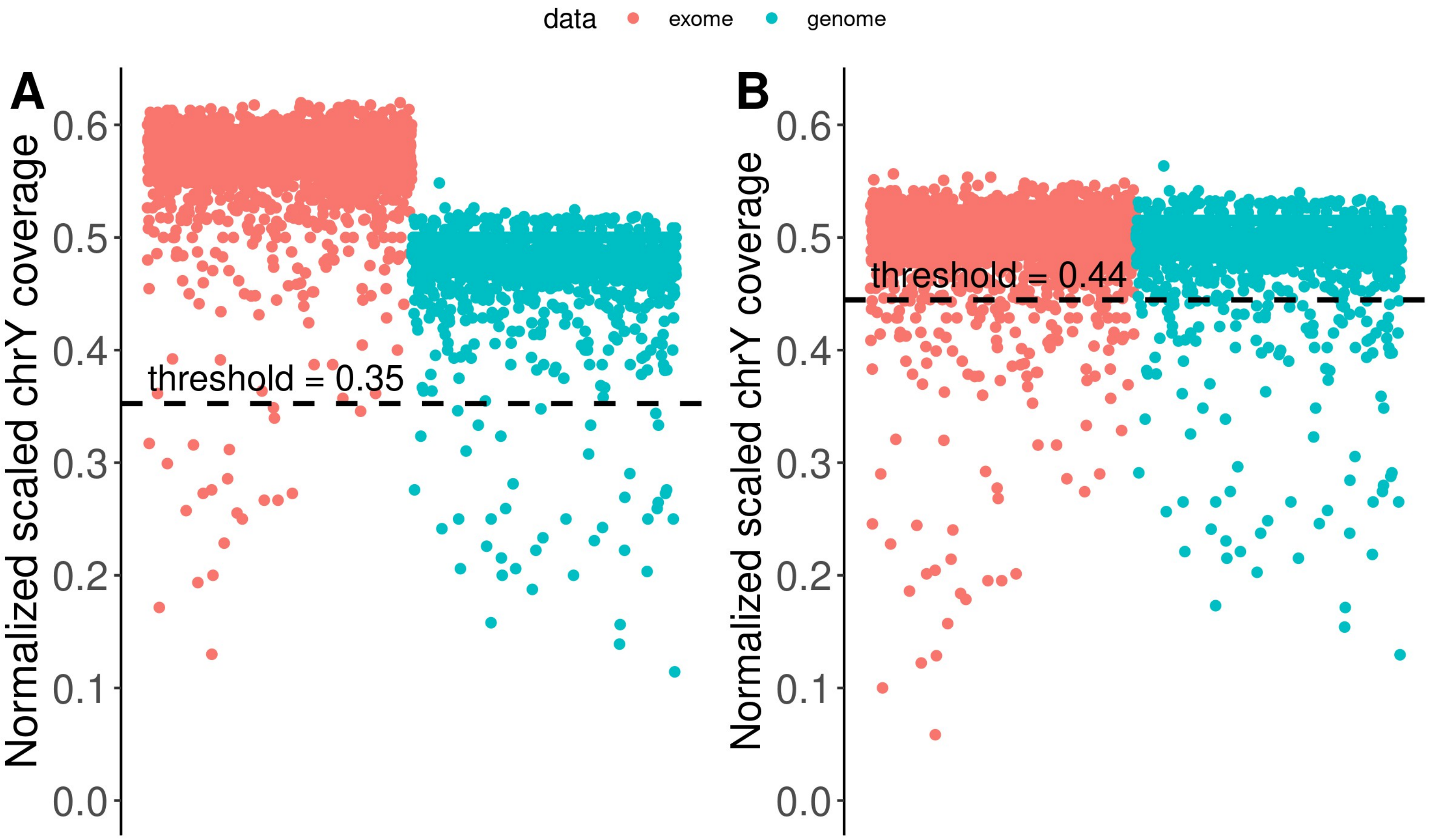
data
exome
genome
A
B
Normalized scaled chrY coverage
0.0
0.1
0.2
0.3
0.4
0.5
0.6
threshold = 0.35
threshold = 0.44

Figure S6. Estimating mLOY with MosCoverY from the mix of exome sequencing and WGS data of 4,200 randomly selected participants from the UKB. A - exome sequencing and WGS data are first combined and then rescaled to the population median normalized chrY coverage of 0.5. B - exome sequencing and WGS data are first rescaled to the population median normalized chrY coverage of 0.5 separately, and then combined to define a binary threshold. In both cases, the threshold is defined as Q1$-$1.5×IQR of the normalized scaled chrY coverage.

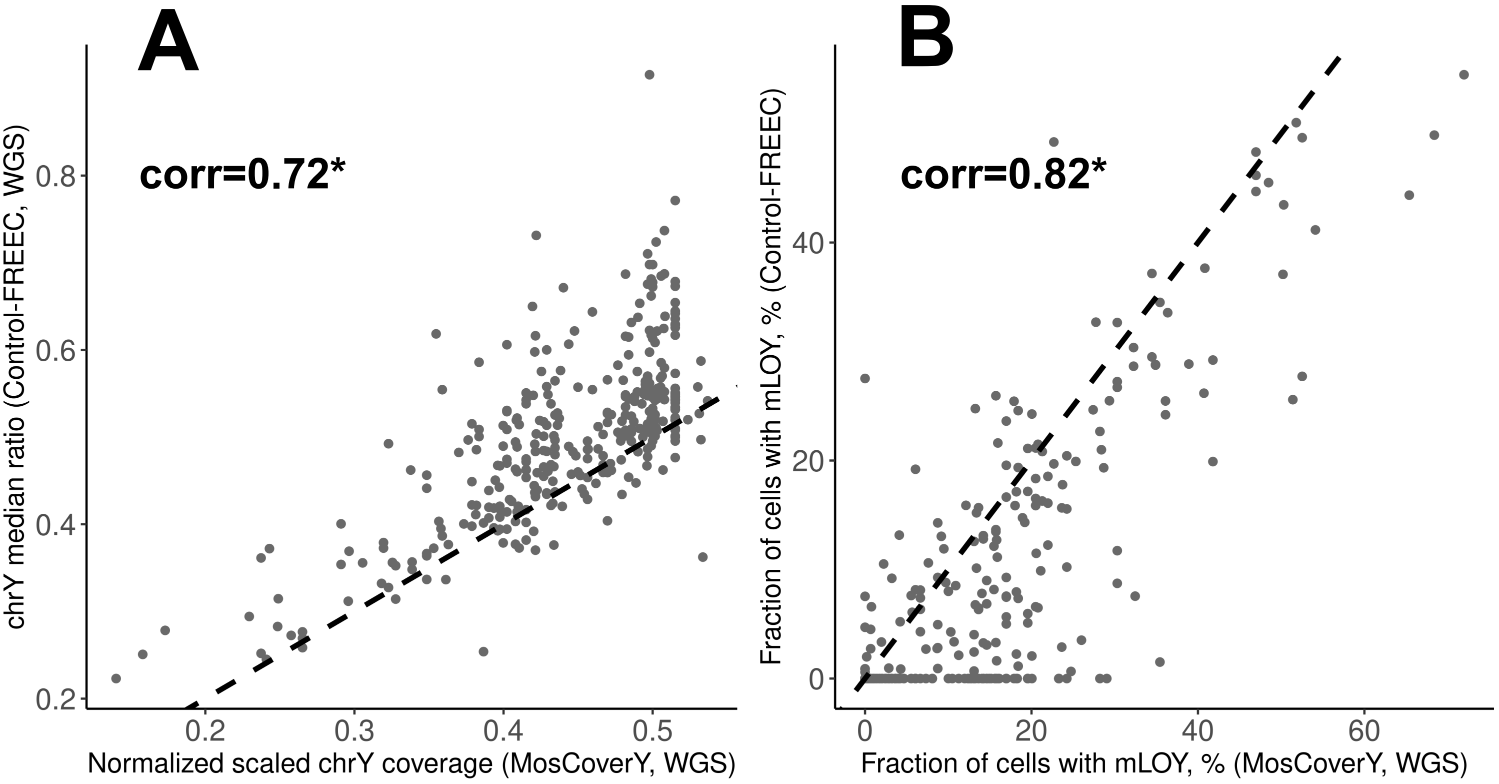
A
corr=0.72*
chrY median ratio (Control-FREEC, WGS)
0.2
0.4
0.6
0.8
0.2
0.3
0.4
0.5
Normalized scaled chrY coverage (MosCoverY, WGS)
B
corr=0.82*
Fraction of cells with mLOY, % (Control-FREEC)
0
20
40
0
20
40
60
Fraction of cells with mLOY, % (MosCoverY, WGS)

Figure S7. Comparison of MosCoverY and Control-FREEC results on WGS data for a subset of 360 randomly selected individuals from the UKB. A - Normalized scaled chrY coverage as estimated by MosCoverY from WGS data vs chrY median ratio (normalized copy number) as estimated by Control-FREEC from WGS data. B - Estimated fraction of cells with mLOY estimated by MosCoverY applied to WGS data and by Control-FREEC, in both cases, Equation 1 was used (corr - Pearson's correlation coefficient, * - p-value < 0.001).

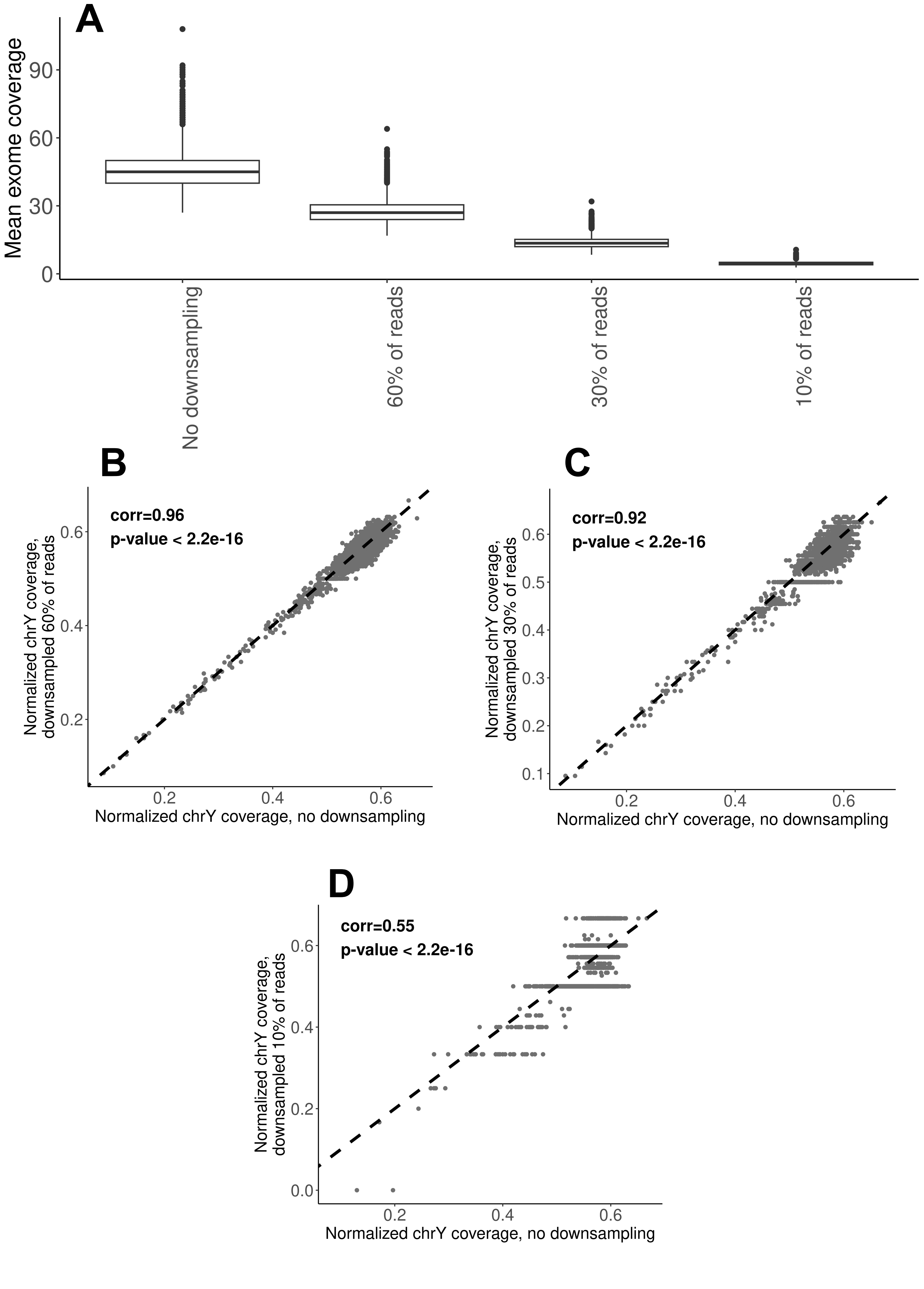

A
Mean exome coverage
0
30
60
90
No downsampling
60% of reads
30% of reads
10% of reads
B
corr=0.96
p-value < 2.2e-16
Normalized chrY coverage, downsampled 60% of reads
Normalized chrY coverage, no downsampling
C
corr=0.92
p-value < 2.2e-16
Normalized chrY coverage, downsampled 30% of reads
Normalized chrY coverage, no downsampling
D
corr=0.55
p-value < 2.2e-16
Normalized chrY coverage, downsampled 10% of reads
Normalized chrY coverage, no downsampling

Figure S8. The effect of different exome coverage on MosCoverY results. MosCoverY was applied to a random subset of 4,200 individuals from the UKB to the original and downsampled at the 60%, 30% and 10% fraction of reads of exome sequencing alignment files (.cram). A - Distribution of mean coverage for each downsampling group. B, C, D - Correlation of normalized chrY coverage estimated with MosCoverY from downsampled cram files with the original cram files, respectively for 60%, 30%, and 10% of reads kept (corr - Pearson’s correlation coefficient).

mLOY estimation method	N with mLOY	HR	p-value
MosCoverY only	1300	1.01	0.82
PAR-LOY only	10511	0.98	0.49
mLRRY only	1125	1.04	0.58
MosCoverY and PAR-LOY	3469	0.98	0.63
MosCoverY and mLRRY	960	1.11	0.11
PAR-LOY and mLRRY	339	0.84	0.21
MosCoverY, PAR-LOY and mLRRY	4611	1.12	0.00047

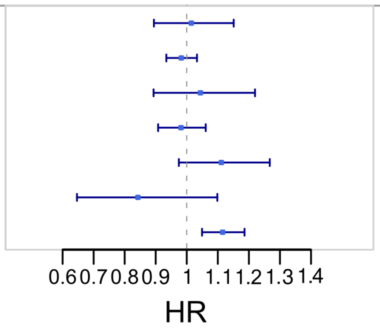

Figure S9. Association of binary mLOY traits defined by different combinations of methods with all-cause mortality estimated by the Cox proportional hazard model adjusted for age, smoking status, and genetic PCs. Groups are defined by mLOY carriers identified by one, two, or all three methods (same way as in Fig. 1C, removing individuals of non-European ancestry). The analysis is performed on individuals of European ancestry. HR - Hazard Ratio.

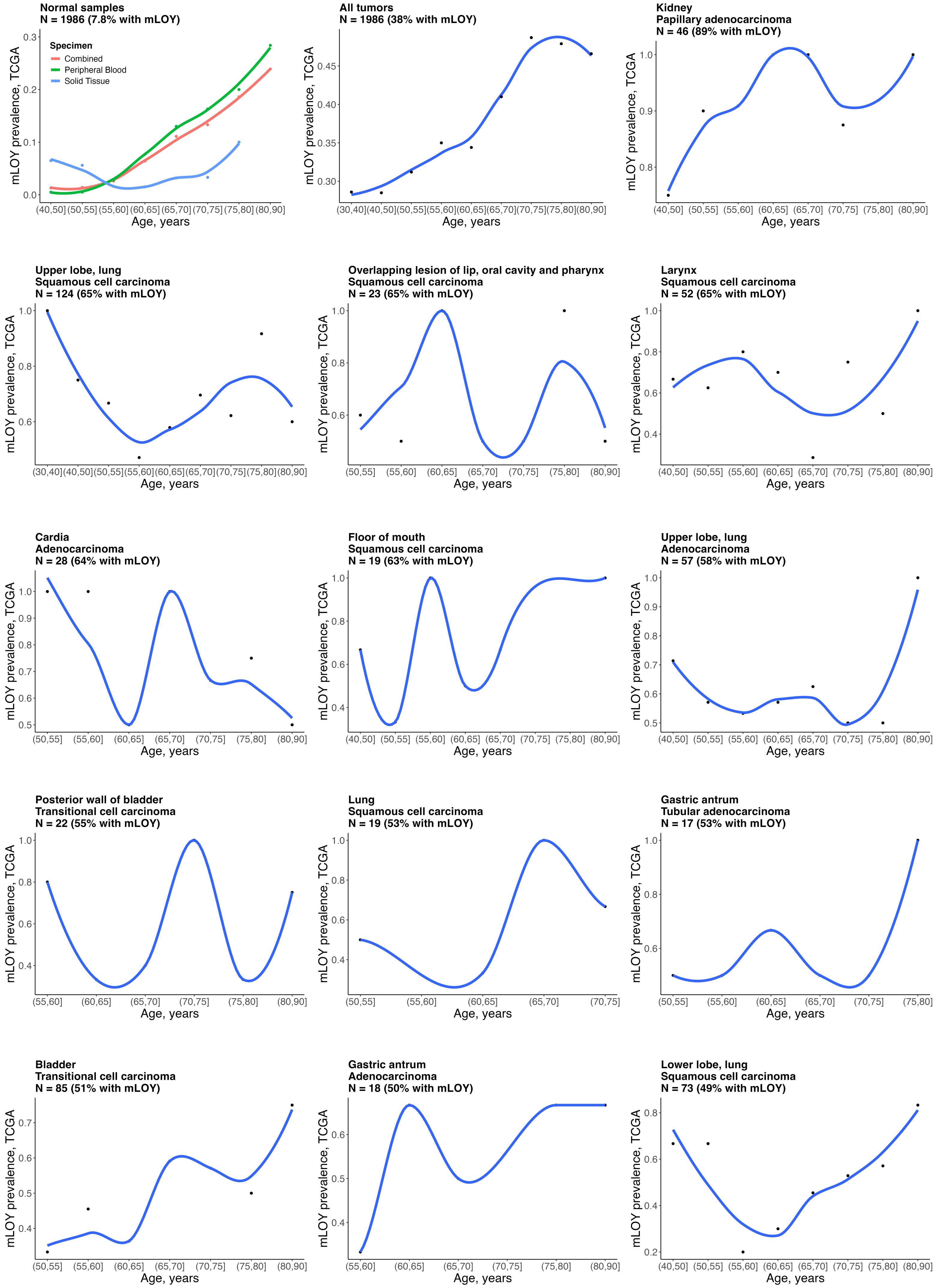
Normal samples
N = 1986 (7.8% with mLOY)
Specimen
Combined
Peripheral Blood
Solid Tissue
All tumors
N = 1986 (38% with mLOY)
Kidney
Papillary adenocarcinoma
N = 46 (89% with mLOY)
Upper lobe, lung
Squamous cell carcinoma
N = 124 (65% with mLOY)
Overlapping lesion of lip, oral cavity and pharynx
Squamous cell carcinoma
N = 23 (65% with mLOY)
Larynx
Squamous cell carcinoma
N = 52 (65% with mLOY)
Cardia
Adenocarcinoma
N = 28 (64% with mLOY)
Floor of mouth
Squamous cell carcinoma
N = 19 (63% with mLOY)
Upper lobe, lung
Adenocarcinoma
N = 57 (58% with mLOY)
Posterior wall of bladder
Transitional cell carcinoma
N = 22 (55% with mLOY)
Lung
Squamous cell carcinoma
N = 19 (53% with mLOY)
Gastric antrum
Tubular adenocarcinoma
N = 17 (53% with mLOY)
Bladder
Transitional cell carcinoma
N = 85 (51% with mLOY)
Gastric antrum
Adenocarcinoma
N = 18 (50% with mLOY)
Lower lobe, lung
Squamous cell carcinoma
N = 73 (49% with mLOY)
mLOY prevalence, TCGA
Age, years

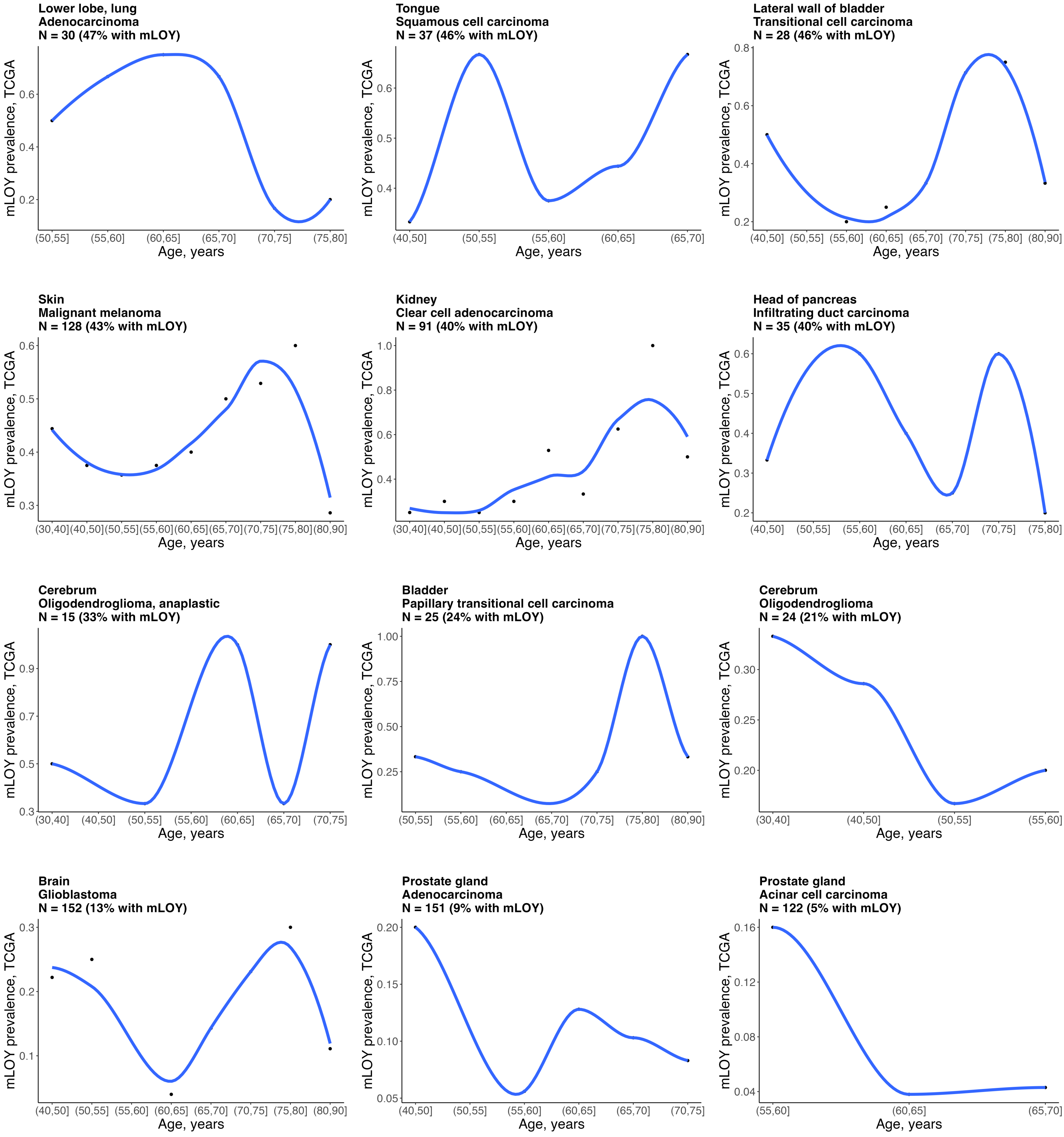

Lower lobe, lung
Adenocarcinoma
N = 30 (47% with mLOY)
mLOY prevalence, TCGA
0.2
0.4
0.6
(50,55]
(55,60]
(60,65]
(65,70]
(70,75]
(75,80]
Age, years
Tongue
Squamous cell carcinoma
N = 37 (46% with mLOY)
mLOY prevalence, TCGA
0.4
0.5
0.6
(40,50]
(50,55]
(55,60]
(60,65]
(65,70]
Age, years
Lateral wall of bladder
Transitional cell carcinoma
N = 28 (46% with mLOY)
mLOY prevalence, TCGA
0.2
0.4
0.6
0.8
(40,50]
(50,55]
(55,60]
(60,65]
(65,70]
(70,75]
(75,80]
(80,90]
Age, years
Skin
Malignant melanoma
N = 128 (43% with mLOY)
mLOY prevalence, TCGA
0.3
0.4
0.5
0.6
(30,40]
(40,50]
(50,55]
(55,60]
(60,65]
(65,70]
(70,75]
(75,80]
(80,90]
Age, years
Kidney
Clear cell adenocarcinoma
N = 91 (40% with mLOY)
mLOY prevalence, TCGA
0.4
0.6
0.8
1.0
(30,40]
(40,50]
(50,55]
(55,60]
(60,65]
(65,70]
(70,75]
(75,80]
(80,90]
Age, years
Head of pancreas
Infiltrating duct carcinoma
N = 35 (40% with mLOY)
mLOY prevalence, TCGA
0.2
0.3
0.4
0.5
0.6
(40,50]
(50,55]
(55,60]
(60,65]
(65,70]
(70,75]
(75,80]
Age, years
Cerebrum
Oligodendroglioma, anaplastic
N = 15 (33% with mLOY)
mLOY prevalence, TCGA
0.3
0.5
0.7
0.9
(30,40]
(40,50]
(50,55]
(55,60]
(60,65]
(65,70]
(70,75]
Age, years
Bladder
Papillary transitional cell carcinoma
N = 25 (24% with mLOY)
mLOY prevalence, TCGA
0.25
0.50
0.75
1.00
(50,55]
(55,60]
(60,65]
(65,70]
(70,75]
(75,80]
(80,90]
Age, years
Cerebrum
Oligodendroglioma
N = 24 (21% with mLOY)
mLOY prevalence, TCGA
0.20
0.25
0.30
(30,40]
(40,50]
(50,55]
(55,60]
Age, years
Brain
Glioblastoma
N = 152 (13% with mLOY)
mLOY prevalence, TCGA
0.1
0.2
0.3
(40,50]
(50,55]
(55,60]
(60,65]
(65,70]
(70,75]
(75,80]
(80,90]
Age, years
Prostate gland
Adenocarcinoma
N = 151 (9% with mLOY)
mLOY prevalence, TCGA
0.05
0.10
0.15
0.20
(40,50]
(50,55]
(55,60]
(60,65]
(65,70]
(70,75]
Age, years
Prostate gland
Acinar cell carcinoma
N = 122 (5% with mLOY)
mLOY prevalence, TCGA
0.04
0.08
0.12
0.16
(55,60]
(60,65]
(65,70]
Age, years

Figure S10. Prevalence of mLOY with age in TCGA in normal (upper left corner) and tumor samples (all tumor types combined and tumor-specific prevalence if the number of individuals with the specific tumor type is at least 15). Age is binned due to the limited number of individuals.

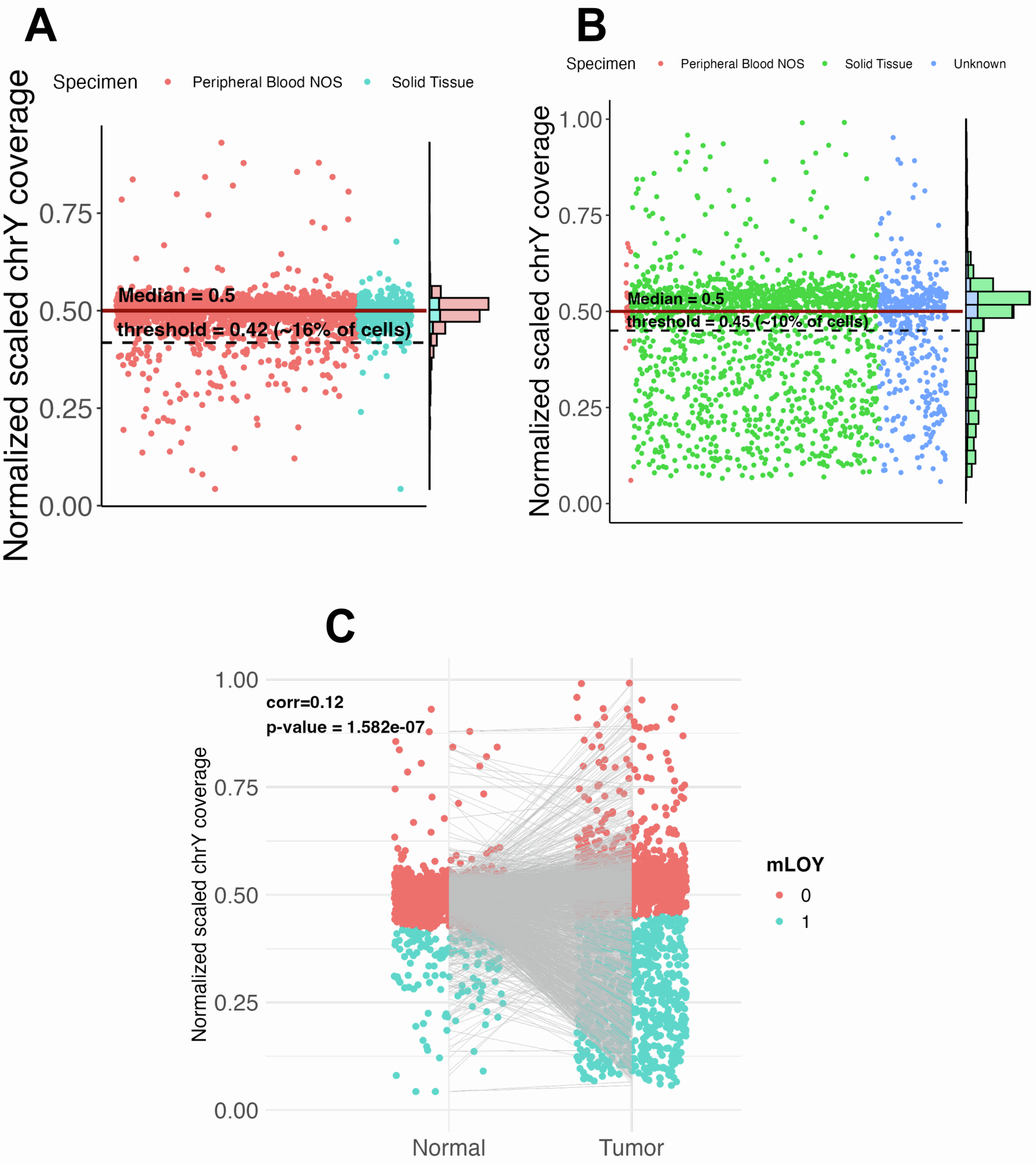

A
Specimen
Peripheral Blood NOS
Solid Tissue
Normalized scaled chrY coverage
0.00
0.25
0.50
0.75
Median = 0.5
threshold = 0.42 (~16% of cells)
B
Specimen
Peripheral Blood NOS
Solid Tissue
Unknown
Normalized scaled chrY coverage
0.00
0.25
0.50
0.75
1.00
Median = 0.5
threshold = 0.45 (~10% of cells)
C
corr=0.12
p-value = 1.582e-07
Normalized scaled chrY coverage
0.00
0.25
0.50
0.75
1.00
Normal
Tumor
mLOY
0
1

Figure S11. Results of MosCoverY on TCGA exome sequencing data. A - Distribution of normalized scaled chrY coverage in normal samples. The dashed line represents a threshold defined as Q1−1.5×IQR of normalized scaled chrY coverage. B - Distribution of normalized scaled chrY coverage in tumor samples. The dashed line represents a threshold arbitrarily defined as 0.45 (~10% of cells with mLOY). C - Correlation of normalized scaled chrY coverage between normal and tumor samples. In all panels depicting normalized scaled chrY coverage values of more than 1 are removed (corr - Pearson’s correlation coefficient).

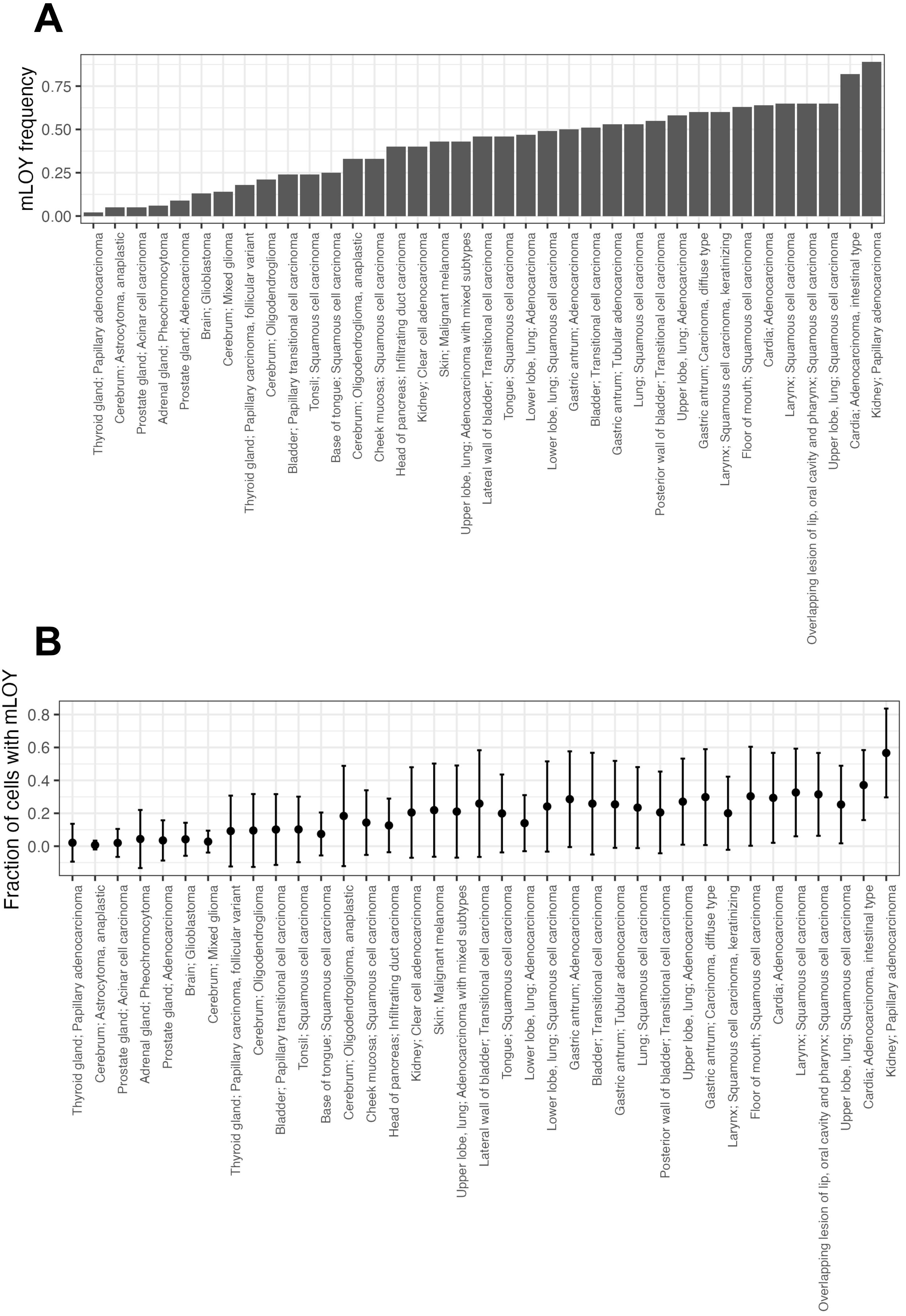

A
mLOY frequency
0.00
0.25
0.50
0.75
Thyroid gland; Papillary adenocarcinoma
Cerebrum; Astrocytoma, anaplastic
Prostate gland; Acinar cell carcinoma
Adrenal gland; Pheochromocytoma
Prostate gland; Adenocarcinoma
Brain; Glioblastoma
Cerebrum; Mixed glioma
Thyroid gland; Papillary carcinoma, follicular variant
Cerebrum; Oligodendroglioma
Bladder; Papillary transitional cell carcinoma
Tonsil; Squamous cell carcinoma
Base of tongue; Squamous cell carcinoma
Cerebrum; Oligodendroglioma, anaplastic
Cheek mucosa; Squamous cell carcinoma
Head of pancreas; Infiltrating duct carcinoma
Kidney; Clear cell adenocarcinoma
Skin; Malignant melanoma
Upper lobe, lung; Adenocarcinoma with mixed subtypes
Lateral wall of bladder; Transitional cell carcinoma
Tongue; Squamous cell carcinoma
Lower lobe, lung; Adenocarcinoma
Lower lobe, lung; Squamous cell carcinoma
Gastric antrum; Adenocarcinoma
Bladder; Transitional cell carcinoma
Gastric antrum; Tubular adenocarcinoma
Lung; Squamous cell carcinoma
Posterior wall of bladder; Transitional cell carcinoma
Upper lobe, lung; Adenocarcinoma
Gastric antrum; Carcinoma, diffuse type
Larynx; Squamous cell carcinoma, keratinizing
Floor of mouth; Squamous cell carcinoma
Cardia; Adenocarcinoma
Larynx; Squamous cell carcinoma
Overlapping lesion of lip, oral cavity and pharynx; Squamous cell carcinoma
Upper lobe, lung; Squamous cell carcinoma
Cardia; Adenocarcinoma, intestinal type
Kidney; Papillary adenocarcinoma
B
Fraction of cells with mLOY
0.0
0.2
0.4
0.6
0.8
Thyroid gland; Papillary adenocarcinoma
Cerebrum; Astrocytoma, anaplastic
Prostate gland; Acinar cell carcinoma
Adrenal gland; Pheochromocytoma
Prostate gland; Adenocarcinoma
Brain; Glioblastoma
Cerebrum; Mixed glioma
Thyroid gland; Papillary carcinoma, follicular variant
Cerebrum; Oligodendroglioma
Bladder; Papillary transitional cell carcinoma
Tonsil; Squamous cell carcinoma
Base of tongue; Squamous cell carcinoma
Cerebrum; Oligodendroglioma, anaplastic
Cheek mucosa; Squamous cell carcinoma
Head of pancreas; Infiltrating duct carcinoma
Kidney; Clear cell adenocarcinoma
Skin; Malignant melanoma
Upper lobe, lung; Adenocarcinoma with mixed subtypes
Lateral wall of bladder; Transitional cell carcinoma
Tongue; Squamous cell carcinoma
Lower lobe, lung; Adenocarcinoma
Lower lobe, lung; Squamous cell carcinoma
Gastric antrum; Adenocarcinoma
Bladder; Transitional cell carcinoma
Gastric antrum; Tubular adenocarcinoma
Lung; Squamous cell carcinoma
Posterior wall of bladder; Transitional cell carcinoma
Upper lobe, lung; Adenocarcinoma
Gastric antrum; Carcinoma, diffuse type
Larynx; Squamous cell carcinoma, keratinizing
Floor of mouth; Squamous cell carcinoma
Cardia; Adenocarcinoma
Larynx; Squamous cell carcinoma
Overlapping lesion of lip, oral cavity and pharynx; Squamous cell carcinoma
Upper lobe, lung; Squamous cell carcinoma
Cardia; Adenocarcinoma, intestinal type
Kidney; Papillary adenocarcinoma

Figure S12. Tumor type-specific frequency and fraction of cells with mLOY. A - binary mLOY frequency in tumor samples by tumor type with at least 10 individuals. B - Mean and standard deviation of the fraction of cells with mLOY in tumor samples by tumor type with at least 10 individuals.

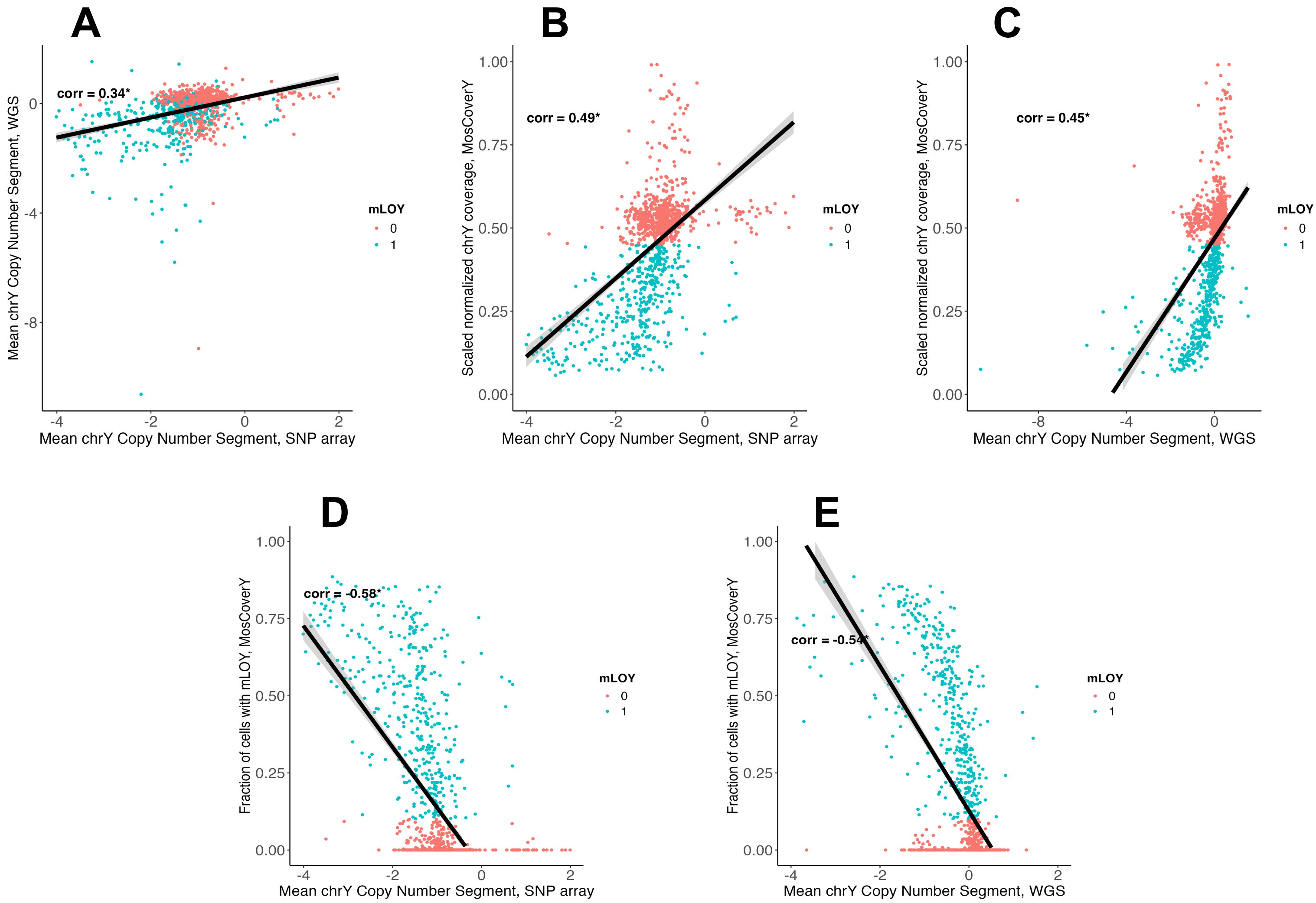

A
corr = 0.34*
Mean chrY Copy Number Segment, WGS
Mean chrY Copy Number Segment, SNP array
mLOY
0
1
B
corr = 0.49*
Scaled normalized chrY coverage, MosCoverY
Mean chrY Copy Number Segment, SNP array
C
corr = 0.45*
Scaled normalized chrY coverage, MosCoverY
Mean chrY Copy Number Segment, WGS
D
corr = -0.58*
Fraction of cells with mLOY, MosCoverY
Mean chrY Copy Number Segment, SNP array
E
corr = -0.54*
Fraction of cells with mLOY, MosCoverY
Mean chrY Copy Number Segment, WGS

Figure S13. Comparison of MosCoverY results applied to exome sequencing data of tumor samples in TCGA with TCGA Copy Number Segment data available that were called from SNP array and WGS. Color represents mLOY carriers identified by MosCoverY. A - TCGA Copy Number Segment data called from WGS, averaged over chrY (y-axis) vs TCGA Copy Number Segment data called from SNP array, averaged over chrY (x-axis). B - Scaled normalized chrY coverage estimated with MosCoverY (y-axis) vs TCGA Copy Number Segment data called from SNP array, averaged over chrY (x-axis). C - Scaled normalized chrY coverage estimated with MosCoverY (y-axis) vs TCGA Copy Number Segment data called from WGS, averaged over chrY (x-axis). D - Fraction of cells with mLOY estimated with MosCoverY (y-axis) vs TCGA Copy Number Segment data called from SNP array, averaged over chrY (x-axis). E - Fraction of cells with mLOY estimated with MosCoverY (y-axis) vs TCGA Copy Number Segment data called from WGS, averaged over chrY (x-axis). corr - Pearson's correlation coefficient, * - p-value < 0.001. The black line represents a linear regression fitted to the points, with shaded areas representing a 95% confidence interval.

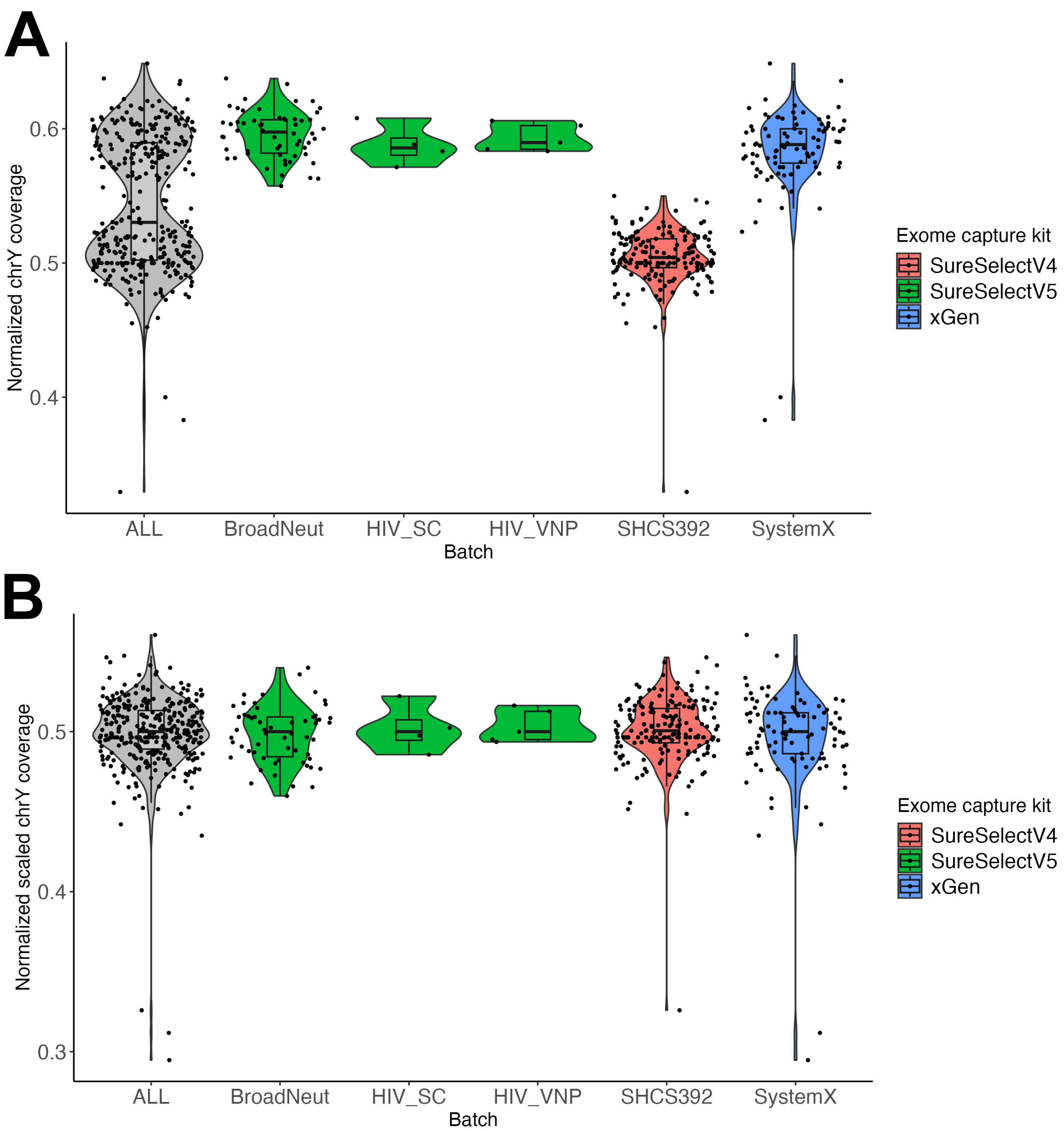
A
Normalized chrY coverage
0.6
0.5
0.4
ALL
BroadNeut
HIV_SC
HIV_VNP
SHCS392
SystemX
Batch
Exome capture kit
SureSelectV4
SureSelectV5
xGen
B
Normalized scaled chrY coverage
0.5
0.4
0.3
ALL
BroadNeut
HIV_SC
HIV_VNP
SHCS392
SystemX
Batch
Exome capture kit
SureSelectV4
SureSelectV5
xGen

Figure S14. Distribution of normalized chrY coverage before (A) and after (B) scaling to the population median of 0.5 in 337 men from SHCS. The exomes were sequenced across 5 batches (x-axis) with three exome capture kits (color). The batch ALL depicted in grey represents all batches pooled together. Scaling (B) was done separately for each exome capture kit.

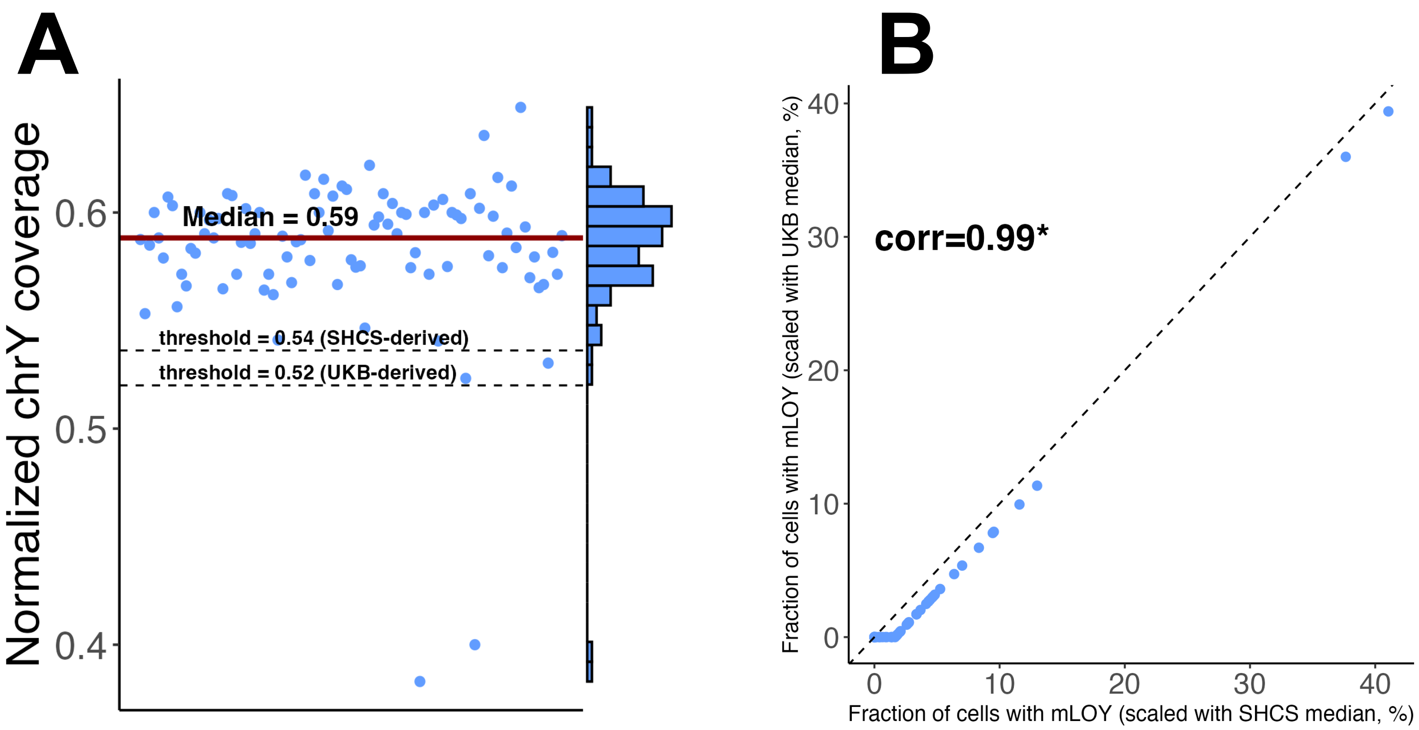
A
Normalized chrY coverage
0.6
0.5
0.4
Median = 0.59
threshold = 0.54 (SHCS-derived)
threshold = 0.52 (UKB-derived)
B
Fraction of cells with mLOY (scaled with UKB median, %)
40
30
20
10
0
corr=0.99*
0
10
20
30
40
Fraction of cells with mLOY (scaled with SHCS median, %)

Figure S15. Comparison of binary mLOY thresholds (A) derived using the UKB median chrY normalized coverage and the SHCS median chrY normalized coverage applied to the SystemX batch of SHCS (sequenced with xGen Exome capture kit, the same as in the UKB), as well as the fraction of cells with mLOY (B) derived using the UKB and the SHCS median of normalized chrY coverage for rescaling normalized chrY coverage to use it as an argument in Equation 1 (corr - Pearson's correlation coefficient, * - p-value < 0.001).

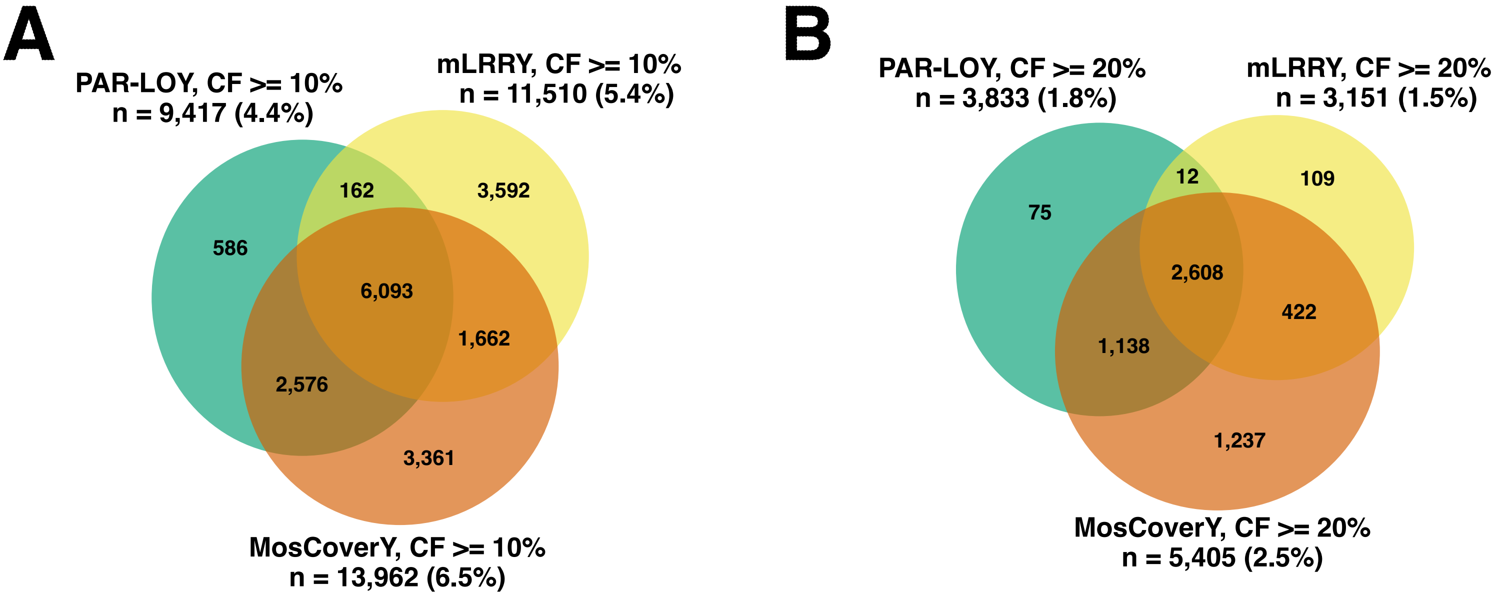
A
PAR-LOY, CF >= 10%
n = 9,417 (4.4%)
mLRRY, CF >= 10%
n = 11,510 (5.4%)
586
162
3,592
6,093
1,662
2,576
3,361
MosCoverY, CF >= 10%
n = 13,962 (6.5%)
B
PAR-LOY, CF >= 20%
n = 3,833 (1.8%)
mLRRY, CF >= 20%
n = 3,151 (1.5%)
75
12
109
2,608
422
1,138
1,237
MosCoverY, CF >= 20%
n = 5,405 (2.5%)

Figure S16. Comparison of different arbitrarily selected thresholds for defining binary mLOY estimates with three methods (PAR-LOY, mLRRY, and MosCoverY) in the UKB. A - Threshold at 10% of cells with LOY; B - Threshold at 20% of cells with LOY.
